# (+)-Rutamarin as a Dual Inducer of Both GLUT4 Translocation and Expression Efficiently Ameliorates Glucose Homeostasis in Insulin-Resistant Mice

**DOI:** 10.1371/journal.pone.0031811

**Published:** 2012-02-27

**Authors:** Yu Zhang, Haitao Zhang, Xin-gang Yao, Hong Shen, Jing Chen, Chenjing Li, Lili Chen, Mingyue Zheng, Jiming Ye, Lihong Hu, Xu Shen, Hualiang Jiang

**Affiliations:** 1 State Key Laboratory of Drug Research, Shanghai Institute of Materia Medica, Chinese Academy of Sciences, Shanghai, China; 2 Molecular Pharmacology for Diabetes, School of Health Sciences, RMIT University, Melbourne, Victoria, Australia; University of Texas Health Science Center at Houston, United States of America

## Abstract

Glucose transporter 4 (GLUT4) is a principal glucose transporter in response to insulin, and impaired translocation or decreased expression of GLUT4 is believed to be one of the major pathological features of type 2 diabetes mellitus (T2DM). Therefore, induction of GLUT4 translocation or/and expression is a promising strategy for anti-T2DM drug discovery. Here we report that the natural product (+)-Rutamarin (Rut) functions as an efficient dual inducer on both insulin-induced GLUT4 translocation and expression. Rut-treated 3T3-L1 adipocytes exhibit efficiently enhanced insulin-induced glucose uptake, while diet-induced obese (DIO) mice based assays further confirm the Rut-induced improvement of glucose homeostasis and insulin sensitivity *in vivo*. Subsequent investigation of Rut acting targets indicates that as a specific protein tyrosine phosphatase 1B (PTP1B) inhibitor Rut induces basal GLUT4 translocation to some extent and largely enhances insulin-induced GLUT4 translocation through PI3 kinase-AKT/PKB pathway, while as an agonist of retinoid X receptor α (RXRα), Rut potently increases GLUT4 expression. Furthermore, by using molecular modeling and crystallographic approaches, the possible binding modes of Rut to these two targets have been also determined at atomic levels. All our results have thus highlighted the potential of Rut as both a valuable lead compound for anti-T2DM drug discovery and a promising chemical probe for GLUT4 associated pathways exploration.

## Introduction

GLUT4 as a member of glucose transporters (Gluts) family plays a critical role in glucose uptake in several insulin target tissues, such as muscle and adipose [1], [2]. The importance of GLUT4 for maintaining glucose homeostasis and insulin sensitivity has been extensively addressed in different animal models [3]–[5]. GLUT4 is exquisitely controlled by its translocation or/and expression in tissue-specific, hormone-regulated, and metabolic manners [6]–[10]. The capability of GLUT4 translocation evaluated by its endocytosis and exocytosis ratio is well regulated in physiological condition [11], and GLUT4 translocation impairment is tightly associated with insulin resistance and elevated of plasma glucose levels [1], [11]. In addition, GLUT4 expression also exhibits its significance in insulin responsiveness and glucose tolerance as indicated in GLUT4 deficient or over-expressed mice model [1].

As reported, dysfunctional glucose uptake in muscle or adipose contributes largely to the onset of T2DM [12]. Considering the vital role of GLUT4 in the rate-limit step of glucose transport, targeting the pathways associated with GLUT4 has thus become an attractive strategy for drug discovery against T2DM and other metabolic disorders [13], [14]. Recent research has shown that the agents capable of regulating GLUT4 translocation or expression may exhibit their potentials for anti-diabetic treatments. Metformin, as a major oral anti-diabetic drug, can efficiently modulate the insulin-mediated GLUT4 translocation [15]. AICAR, which improves glucose homeostasis in *ob/ob* mice, could increase GLUT4 mRNA/protein expression levels [16], [17]. Since induction of GLUT4 translocation or expression has been proved to be effective for anti-T2DM therapy, the agents with dual functions in enhancing both GLUT4 translocation and expression are expected to exhibit more potent anti-diabetes properties. Moreover, either GLUT4 translocation or expression is involved in complicated pathways, which are still poorly understood. Dual-functional agents could be good probes for unraveling such pathways. Accordingly, we seek to discover the agents that potently induce both GLUT4 translocation and expression.

**Figure 1:**
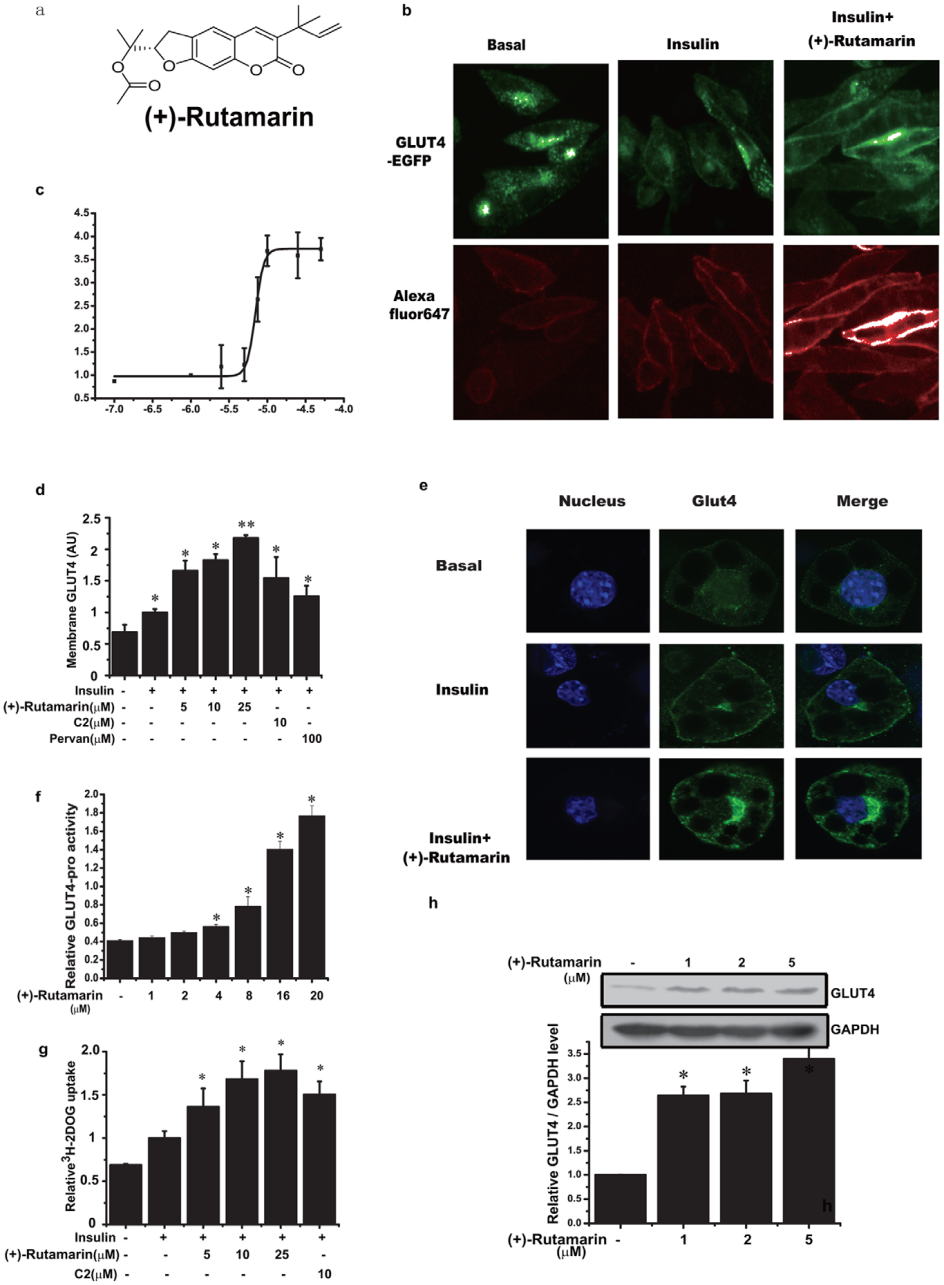
(+)-Rutamarin enhances both GLUT4 translocation and expression. (**a**) Chemical structure of (+)-Rutamarin. (**b**) Insulin (170 nM) activates GLUT4 translocation within 5 minutes in CHO-K1/GLUT4 cells that are pre-treated with Rut (20 µM) for 8 hours followed by insulin (170 nM) stimulation for 5 minutes, with (**c**) EC_50_ value of 7.0 µM. Green fluorescence represents the total GLUT4, and red fluorescence depicts the translocated GLUT4 to cell membrane. (**d**) Insulin (170 nM)-induced GLUT4 translocation of CHO-K1/GLUT4 cells treated with compound-2 (C2, 10 µM), pervanadate (Pervan, 100 µM) and Rut (5, 10, 20 µM) for 5 minutes. (**e**) Immunofluorescence with antibody against GLUT4 (green), DAPI (blue) in 3T3-L1 adipocytes. GLUT4 translocation is detected either in the insulin (17 nM) stimulated 3T3-L1 adipocytes for 5 minutes, or pre-treated with Rut (20 µM) for 8 hours and then stimulated with insulin (17 nM) for 5 minutes. (**f**) Rut potently stimulates GLUT4 promoter activity in 3T3-L1 adipocytes (20 µM, Rut was pre-treated in the cells for 24 hours. Reporter gene is GLUT4 promoter-luciferase plasmid together with Renilla luciferase expression vector as a control). (**g**) H^3^-glucose uptake assay in fully differentiated 3T3-L1 adipocytes treated with C2 (10 µM) and different doses of Rut (5–25 µM). Treatment of Rut for 30 minutes improves the insulin (17 nM)-stimulated glucose uptake. (**h**) GLUT4 expression is determined by western blotting with an antibody against GLUT4 in 3T3-L1 adipocytes treated with Rut (20 µM) for 48 hours. Data are presented as means ± s.e.m (*P<0.05, **P<0.01) from three independent experiments.

Considering that natural products are major resources of bioactive agents for their large-scale structural diversity, we have screened the in-house natural product library (∼5000 compounds) by the constructed IN Cell 1000 Analyzer based high-content-screening platform. Finally, we determined the natural product (+)-Rutamarin (Rut, **Fig. 1a**) that potently induces both GLUT4 translocation and expression. Rut can efficiently improve insulin sensitivity and glucose homeostasis in diet-induced obese (DIO) mice. Further research indicates that Rut takes its effects by acting on two distinct targets. As a specific inhibitor of protein tyrosine phosphatase 1B (PTP 1B), it stimulates GLUT4 translocation and glucose uptake, while as an RXRα agonist Rut enhances GLUT4 expression in 3T3-L1 adipocytes. Moreover, using molecular modeling and crystallographic approaches, the binding of Rut against these two targets has been fully investigated at atomic levels. Our results have thus highlighted the potential for this natural product as a promising lead compound in anti-T2MD drug discovery, and as a valuable chemical probe in GLUT4 complicated pathways investigation.

## Results

### (+)-Rutamarin (Rut) is a dual inducer of both GLUT4 translocation and expression

Based on the lab-constructed IN Cell Analyzer 1000 platform (see Experimental Procedures) against the in-house natural product library (∼5000 compounds), we have discovered 10 natural products that strongly induce the insulin-stimulated GLUT4 translocation. Among these compounds, Rut exhibits its stimulation activity of EC_50_ at 7.0 µM (**Fig. 1b, c**). Interestingly, the enhancement of Rut on the insulin-induced GLUT4 translocation is even stronger than those of compound-2 (C-2), a known PTP1B selective inhibitor [18], and pervanadate, the reported general tyrosine phosphatase inhibitor [19] (**Fig. 1d**). We also find that Rut significantly induces GLUT4 translocation in fully differentiated 3T3-L1 adipocytes (**Fig. 1e**). It is noted that the displayed GLUT4 is the total cellular GLUT4 in **Fig. 1e**. As described in the experimental procedures, before staining with fluorescence antibody, 0.2% Triton X-100 is applied to punching holes in the cell membranes to let antibodies enter cytoplasm. We also notice that the GLUT4 fluorescence intensity is brighter than the control fluorescence intensity (**Fig. 1e**). To study whether insulin or insulin plus Rut treated 3T3-L1 adipocyte might increase GLUT4 protein level in a short time, Western blot is performed. The result shows that GLUT4 protein level keeps no change within the different treatments (Supplemental **Fig. S1a**). As indicated also in **Fig. 1e**, at basal, GLUT4 vesicles are widely separated in cytoplasm, after staining with fluorescence antibody, the whole cell shows similar but weak fluorescence intensity. After insulin or insulin plus Rut stimulation, GLUT4 vesicles are aggregated at the cell membrane near to the cell nucleus. From the above, we conclude that there is no change for the total GLUT4 protein, while the aggregated GLUT4 proteins in different areas made the different fluorescence intensity. As a result, we think that this phenomenon is caused by the accumulation of GLUT4 vesicles or probably due to a change in accessibility of the antigen by the antibody (fixed cells), caused by Rut. In addition, the insulin-stimulated GLUT4 translocation renders around 1.5-fold over the basal in IN Cell 1000 analyzer based system. Such a lower response compared with the published result might be tentatively resulted from the different systems used [20].

Subsequently, all determined compounds with the ability to induce GLUT4 translocation are tested for their induction on GLUT4 expression in 3T3-L1 adipocytes. Among them, only Rut is found to potently increase GLUT4 protein level (**Fig. 1h**) and dose-dependently induce GLUT4 promoter activity (**Fig. 1f**). In investigation of the short or long-term effects of Rut in 3T3-L1 adipocytes, we have detected the insulin-induced GLUT4 translocation and glucose uptake in a time-dependent manner. As indicated, Rut greatly enhances the insulin-induced GLUT4 translocation at 8th hour (Supplemental **Fig. S1c** and **d**) and exhibits no obvious increment on GLUT4 protein level (Supplemental **Fig. S1e**). However, Rut potently increases GLUT4 protein level at 48th hour (**Fig. 1h**). Therefore, Rut is determined to be a dual-functional inducer enhancing the insulin-induced GLUT4 translocation at short term and GLUT4 expression at long term.

As reported, GLUT4 is primarily expressed in muscle and adipose tissues [21]. In response to insulin, both GLUT4 translocation from cytoplasm to cell membrane and GLUT4 expression facilitate glucose transport [1]. Since Rut is capable of inducing both these two processes, it should stimulate the glucose uptake. As expected, Rut dose-dependently enhances the insulin-induced glucose uptake, and such an enhancement is much greater than that of compound-2 (**Fig. 1g**).

### (+)-Rutamarin (Rut) induces GLUT4 translocation as a PTP1B inhibitor

**Figure 2:**
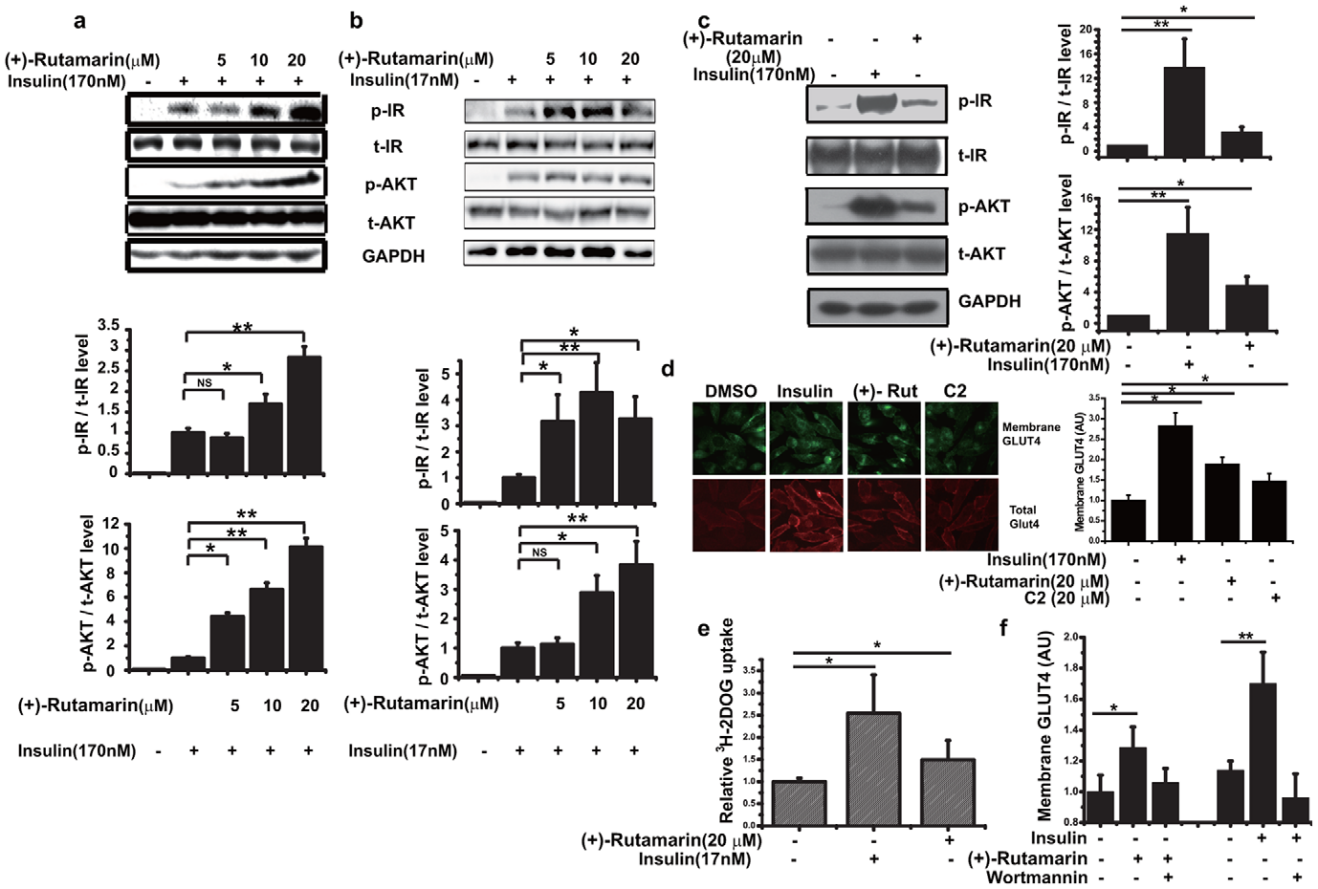
(+)-Rutamarin activates and sensitizes insulin signaling. Phosphorylations of IR and AKT in insulin (170 nM)-stimulated (**a**) CHO-K1 cells and (**b**) insulin (17 nM)-stimulated differentiated 3T3-L1 adipocytes cells for 5 minutes with different doses of Rut determined by western blotting with antibodies against IR (Tyr1146), AKT (Ser473). (**c**) Rut (20 µM) 8 h-treatment increases IR and AKT phosphorylations in CHO-K1 cells. (**d**) Rut (20 µM) 8 h-treatment promotes GLUT4 translocation in CHO-K1/GLUT4 cells. (**e**) Treatment of Rut (20 µM) for 8 hours improves glucose uptake in 3T3-L1 adipocytes without insulin stimulation. (**f**) Wortmannin (1 µM), inhibitor of PI3 kinase, blocks the Rut (20 µM) or insulin (170 nM)-induced GLUT4 translocation. Data are presented as means ± s.e.m (*P<0.05,**P<0.01) from three independent experiments.

To address how Rut takes its effect on GLUT4 translocation, we have investigated the Rut-induced GLUT4-relevant pathways. In the assay, we first confirm the insulin sensitivity of CHO/GLUT4 cell line by Western blot, although there has been already the report on the double stable transfected HEK293 (GLUT4 plus IRS1) based approach [22]. In our work, the robust insulin-induced phosphorylation of insulin receptor (IR) or AKT (**Fig. 2a,c**) has indicated that our platform is reliable for the insulin-stimulated GLUT4 translocation assay.

PI3 kinase-AKT/PKB pathway is confirmed to be involved in the insulin-stimulated GLUT4 translocation [1]. Here we find that, in the insulin-treated CHO-K1 cells, Rut enhances the insulin-induced tyrosine phosphorylation of IR and the serine phosphorylation of AKT in a dose-dependent manner, without altering the total protein levels of IR and AKT (**Fig. 2a**). To further confirm our results in CHO cells, we also use 3T3-L1 adipocytes as a model to study insulin action and glucose metabolism [20]. As indicated, the insulin-stimulated phosphorylation levels of IR and AKT have been largely increased with Rut treatment in 3T3-L1 adipocytes (**Fig. 2b**).

Based on the finding that Rut sensitizes insulin signaling, we next explore the effects of this compound on insulin pathway. As shown in **Fig. 2c**, the phosphorylation levels of IR and AKT are obviously increased in the Rut-treated CHO-K1 cells. Moreover, Rut also induces basal GLUT4 translocation and glucose uptake in CHO-K1 and 3T3-L1 adipocytes without insulin induction (**Fig. 2d, e**). These results thereby suggest that Rut alone can activate insulin signaling, although its effect on glucose uptake is much lower than that of insulin. Therefore, the enhancement of the insulin-induced GLUT4 translocation by Rut has revealed its potent activity in insulin-sensitivity improvement.

To investigate whether PI3 kinase-AKT/PKB pathway is necessary for Rut-induced GLUT4 translocation, the effects of Rut on GLUT4 translocation is detected in the presence or absence of the potent PI3 kinase inhibitor wortmannin. As indicated, wortmannin completely inhibits Rut or insulin-simulated GLUT4 translocation (**Fig. 2f**), implying that PI3 kinase-AKT/PKB pathway is necessary for Rut-induced GLUT4 translocation.

**Figure 3:**
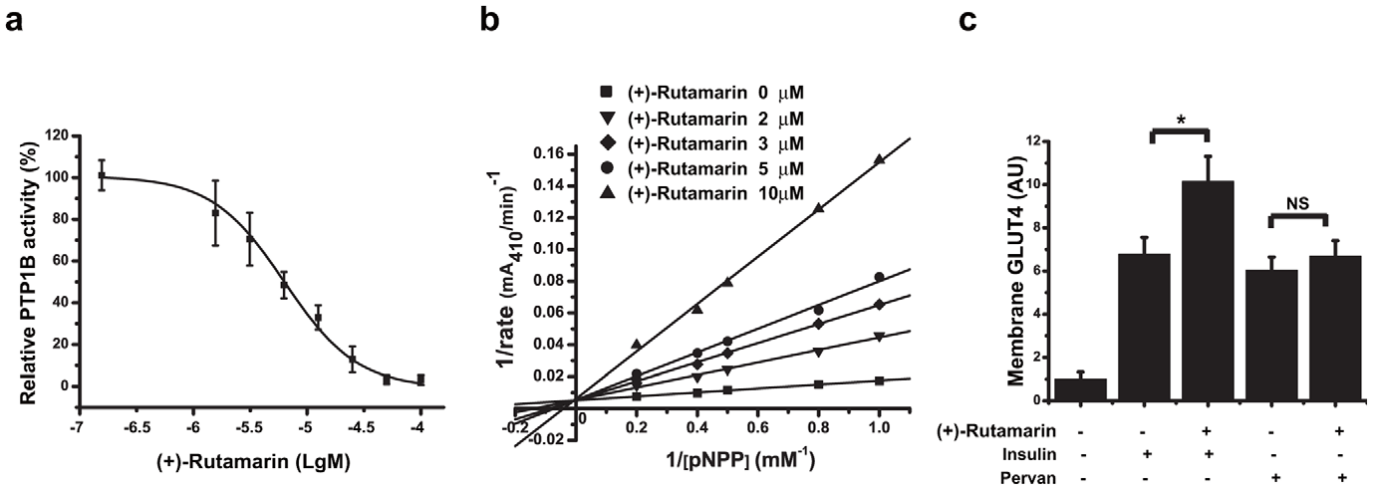
(+)-Rutamarin is a PTP1B inhibitor. (**a**) Rut inhibits the purified PTP1B enzyme activity with IC_50_ value of 6.4 µM. (**b**) The double-reciprocal plots indicates that Rut is a competitive inhibitor of PTP1B determined by Lineweaver-Burk method. (**c**) Rut (20 µM) enhances the insulin but not the pervanadate (100 µM)-induced GLUT4 translocation in CHO/GLUT4 cells. Data are presented as means ± s.e.m (*P<0.05, **P<0.01) from three independent experiments.

Given that Rut can enhance insulin-stimulated IR phosphorylation and its effect on GLUT4 translocation is abolished by wortmannin, it is thus proposed that Rut possibly targets the upstream of AKT, which can also modulate IR phosphorylation. According to our knowledge that the protein tyrosine phosphatases (PTPs) family members can directly dephosphorylate IR thus negatively regulating insulin signaling [23], the PTPs could be one of the first candidates for Rut acting target. To test this hypothesis, the inhibitory activity and selectivity of Rut against PTPs, including PTP1B, PTPα, TC-PTP, LAR and CD45, have been determined. The results are listed in **Table 1**. Rut is determined to be a PTP1B inhibitor (IC_50_ = 6.4 µM, **Fig. 3a**) and shows good selectivity on PTP1B over other PTPs family members (**Table 1**). Moreover, the Lineweaver-Burk analysis indicates that Rut is a competitive inhibitor of PTP1B (**Fig. 3b**).

**Table 1:** Selectivity of compounds against a panel of PTPs.

PTPs	IC_50_ (µM)		
	(+)-Rutamarin	Compound-2	Pervanadate
PTP1B	6.4	5.5	0.015
PTPα	NI^*^	NI^*^	0.059
LAR	NI^*^	NI^*^	0.026
CD45	>100	NI^*^	0.006
TC-PTP	26.8	33.9	0.036

* NI: No inhibition at 100 µM.

To further confirm that Rut induces GLUT4 translocation by inhibiting PTP1B, we test the potential activity of Rut on GLUT4 translocation in the presence or absence of the known PTP1B general inhibitor pervanadate (**Fig. 3c**). As indicated, both Rut and pervanadate induce GLUT4 translocation either with (**Fig. 1d**, and Supplementary **Fig. S1b**) or without insulin stimulation (**Fig. 2e**), while Rut cannot enhance the pervanadate-stimulated GLUT4 translocation, and *vice versa* (**Fig. 3c**), this result thus supports that Rut targets PTP-1B to promote GLUT4 translocation without affecting other pathways.

Therefore, combining the previous results that both pervanadate and compound-2 increase the insulin-stimulated glucose uptake similar to Rut (**Fig. 1d,g**), it is thus suggested that the Rut-induced GLUT4 translocation is through its inhibition of PTP1B.

### (+)-Rutamarin (Rut) stimulates GLUT4 expression as an RXRα agonist

**Figure 4:**
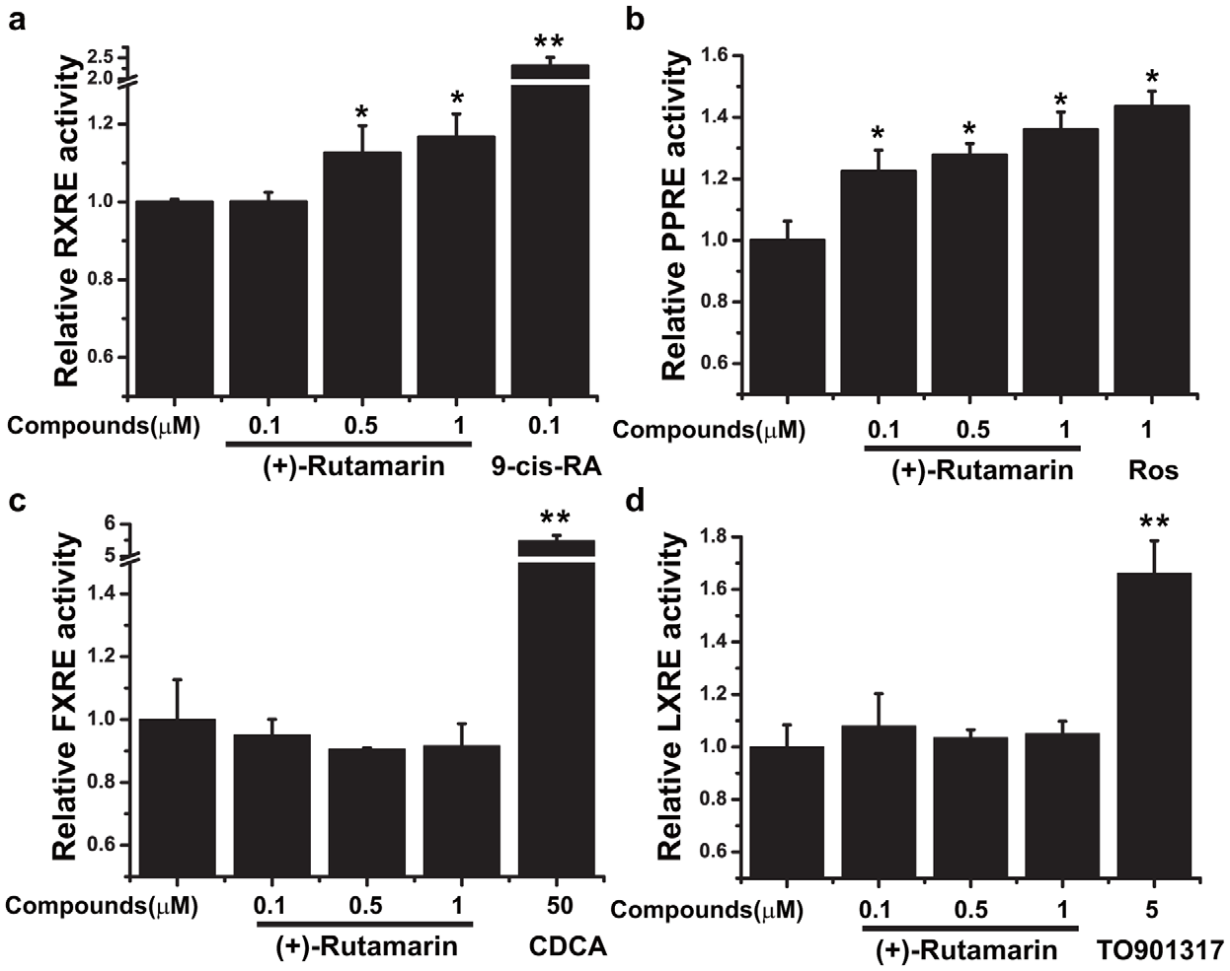
(+)-Rutamarin selectively activates RXRα:RXRα and PPARγ:RXRα transcriptional activities. HEK293 cells are transiently transfected by Lipo2000 respectively with RXRα, PPARγ, FXR, and LXRα plasmids plus pGL3-RXRE luciferase vector and renilla luciferase vector for 5 hours. Cells are then incubated with indicated concentrations of compounds for another 24 h and finally lysed. Luciferase activities are measured using Dual Luciferase Assay System kit. Rut dose-dependently activates (**a**) RXRα:RXRα and (**b**) PPARγ:RXRα activities. Rut has no effects on (**c**) FXR: RXRα and (**d**) LXRα:RXRα activities. Data are presented as means ± s.e.m (*P<0.05, **P<0.01) from three independent experiments.

As demonstrated above, Rut induces GLUT4 promoter activity thereby enhancing GLUT4 expression, while GLUT4 expression has been reported to be regulated by nuclear receptor family, especially PPARγ (Peroxisome proliferator-activated receptor γ) [2]. Accordingly, the effects of Rut on a series of nuclear receptors are determined, including RXRα (Retinoid X receptor α), FXR (Farnesoid X receptor), LXRα (Liver X receptor α) and PPARγ. The results indicate that Rut is able to dose-dependently enhance both the activities of RXR-response element (RXRE) and PPAR-response element (PPRE) (**Fig. 4a, b**) without influencing the activities of LXR-response element (LXRE) and FXR-response element (FXRE) (**Fig. 4c, d**).

**Figure 5:**
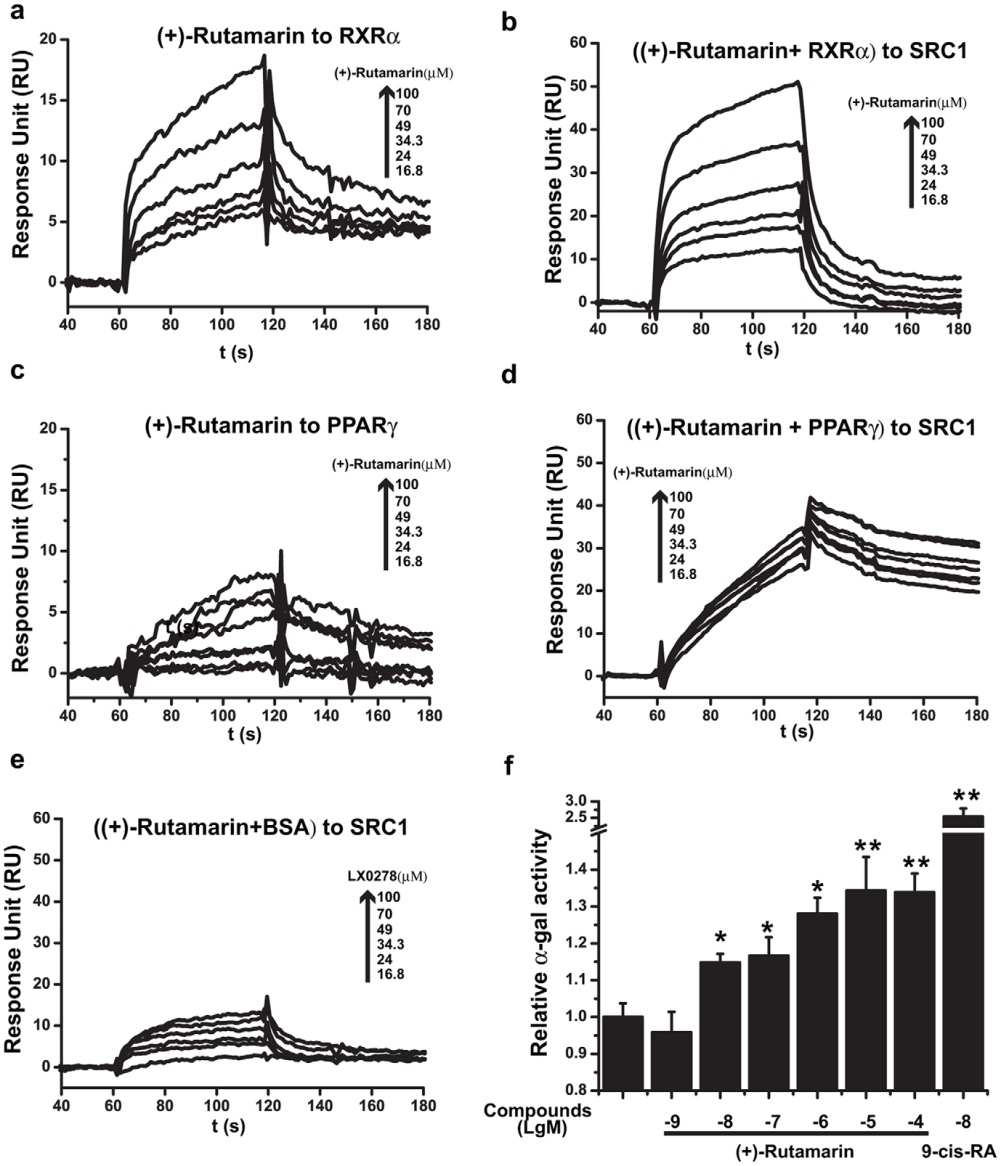
(+)-Rutamarin functions as an RXRα agonist. In SPR technology based Biacore experiment, the purified RXRα-LBD and PPARγ-LBD proteins are immobilized on M5 chips. Rut dose-dependently binds to (**a**) RXRα-LBD but renders no affinity to (**b**) PPARγ-LBD. (**c**) Rut promotes RXRα-LBD/SRC1_613–773_ interaction. (**d**) Rut has few effects on PPARγ-LBD/SRC1_613–773_ interaction (**e**) Rut has no obvious binding affinity to SRC1_613–773_. (**f**) Rut dose-dependently enhances the interaction of RXRα with SRC1 in yeast two-hybrid assay. Data are presented as means ± s.e.m (*P<0.05, **P<0.01) from three independent experiments.

Given that PPARγ partners with RXRα to form a “permissive” heterodimer, which can be activated by either RXRα or PPARγ modulator [24], it is possible that Rut increases the transcriptional activities of both RXRα:RXRα homodimer and PPARγ:RXRα heterodimer by activating RXRα or PPARγ. Therefore, we next test the effects of Rut on the transcriptional activities of RXRα and PPARγ. Firstly, the binding affinity of Rut to the ligand-binding domain (LBD) of RXRα or PPARγ is determined using the surface plasmon resonance (SPR) technology based Biacore instrument. The results show that Rut can dose-dependently bind to RXRα-LBD (**Fig. 5a**) with equilibrium disassociation constant (*K*_D_) value of 5.08 µM (Supplementary **Table S1**). However, there is no binding between Rut and PPARγ-LBD (**Fig. 5b**). As positive controls, the *K*_D_ values of 9-*cis*-retinoic acid (the known RXRα agonist) to RXRα-LBD and rosiglitazone (the known PPARγ agonist) to PPARγ-LBD are determined as 0.66 and 5.82 µM, respectively (Supplementary **Fig. S2a and d, Table S1**). RXRα-LBD or PPARγ-LBD is reported to exhibit notably conformational changes upon agonist binding, subsequently recruiting its co-activator such as the steroid receptor coinducer-1 (SRC1) [25]. To study whether Rut is able to enhance the interaction between RXRα-LBD or PPARγ-LBD and the co-activator, the binding affinity of RXRα-LBD or PPARγ-LBD against SRC1_613–773_ containing the LXXLL motifs [26] in the presence of different concentrations of Rut is measured. As indicated, RXRα-LBD dose-dependently interacts with SRC1 (*K*_D_ = 1.16 µM, Supplementary **Fig. S2b**). Similar to 9-*cis*-retinoic acid (Supplementary **Fig. S2c**), Rut efficiently enhances the interaction between RXRα-LBD and SRC1 (**Fig. 5c**). However, as shown in **Fig. 5d**, Rut cannot enhance the interaction between PPARγ-LBD and SRC1 despite that rosiglitazone greatly enhanced the interaction between PPARγ-LBD and SRC1 (Supplementary **Fig. 2d, e, f**). Additionally, the control experiment shows that Rut does not bind to SRC1 (**Fig. 5e**).

Subsequently, yeast two-hybrid assay is employed to further validate the effect of Rut on the co-activator recruitment. The results clearly show that Rut dose-dependently enhances the interaction of SRC1 with RXRα (**Fig. 5f**). Therefore, based on the SPR and yeast two-hybrid results, we thus conclude that Rut functions as an RXRα agonist (**Fig. 4b**).

**Figure 6:**
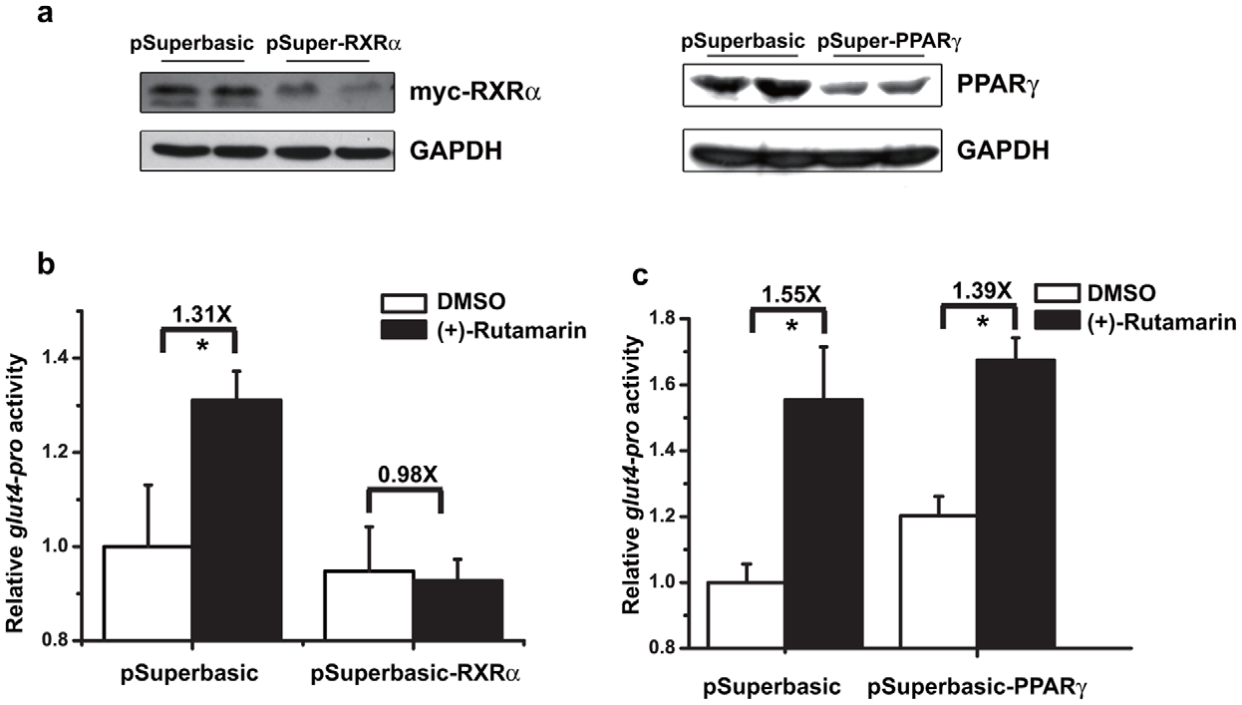
(+)-Rutamarin increases GLUT4 promoter activity through RXRα. (**a**) Knockdown plasmid pSuper-RXRα or pSuper-PPARγ is transiently transfected into HEK293 cells by Lipo2000 for 5 hours, and the knockdown efficiency is determined by western bloting with antibody against RXRα or PPARγ. (**b**) In the presence of *rxrα* siRNA, Rut (20 µM) loses its ability to activate GLUT4 promoter, while similar activation of the promoter is found when pSuperbasic control vector is co-transfected. (**c**) *pparγ* siRNA has no effects on the activation of GLUT4 promoter by Rut (20 µM). Data are presented as means ± s.e.m (*P<0.05,**P<0.01) from three independent experiments.

Considering that RXRα agonist 9-*cis*-retinoic acid could modulate GLUT4 promoter activity thereby increasing GLUT expression [7], [10], we then test whether Rut is also able to increase GLUT4 promoter activity through targeting RXRα. In the assay, the *rxrα*, and *pparγ* gene-specific siRNAs are proved to efficiently reduce transcription of these genes (**Fig. 6a**). In the presence of *rxrα* siRNA, Rut loses its ability to activate GLUT4 promoter, compared with the pSuperbasic control vector assay (**Fig. 6b**). However, *pparγ* siRNA has no effects on Rut-induced GLUT4 promoter activation (**Fig. 6c**), while *pparγ* siRNA itself moderately activated GLUT4 promoter as previously reported [7]. Therefore, our results thus demonstrate that knockdown of RXRα abolishes the Rut-induced increase in GLUT4 promoter activity, thereby suggesting that Rut induces GLUT4 expression by targeting RXRα.

### Binding models of (+)-Rutamarin (Rut) with PTP1B and RXRα

**Figure 7:**
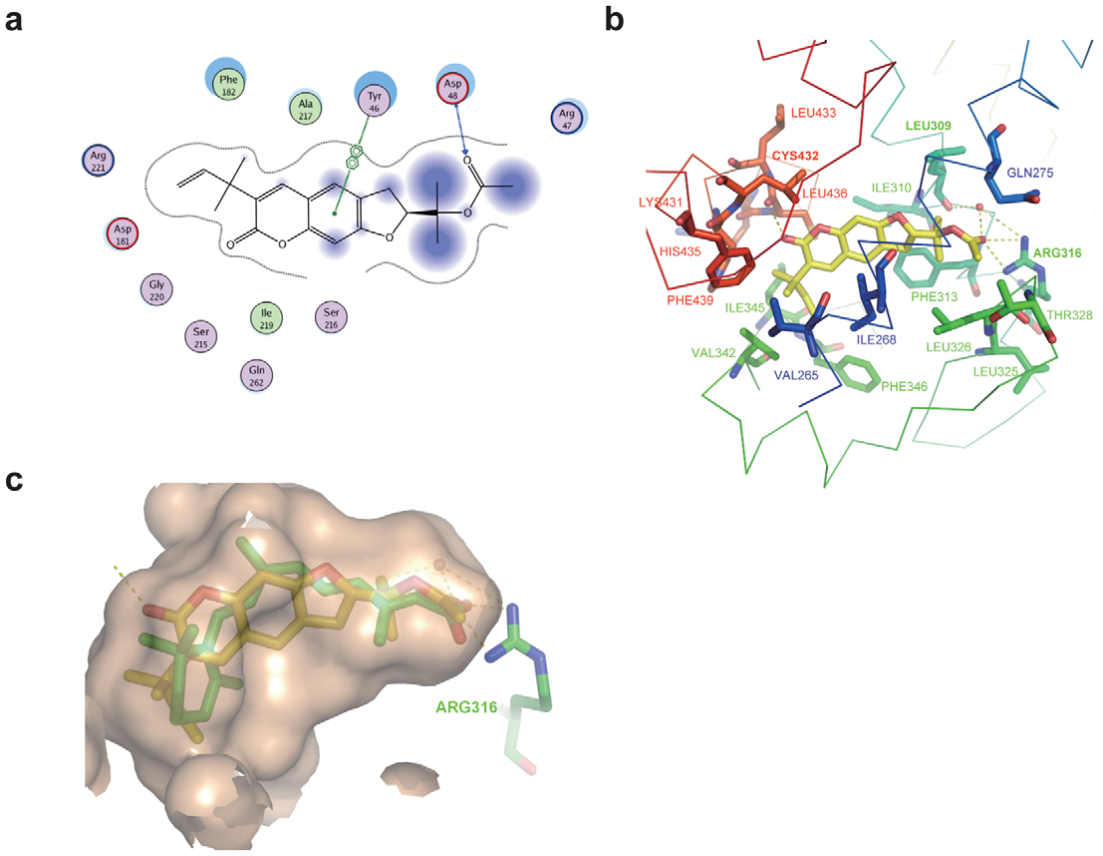
Binding models of (+)-Rutamarin with PTP1B and RXRα. (**a**) The interactions between Rut and the residues in PTP1B substrate-binding site. (**b**) The interactions between Rut and RXRα determined by the crystal structure of RXRα-LBD complexed with Rut. (RXRα is shown as ribbon. All the residues interacting with Rut are shown as sticks. Hydrogen bonds are shown in yellow dot line.) (**c**) Superposition of Rut (yellow) with 9-*cis*-retinoic acid (green) in the ligand-binding pocket, and hydrogen bonds form for these two compounds with residue of Arg316.

Since we failed to obtain the crystal of PTP1B-Rut complex, molecular modeling is thereby performed to investigate the potential binding mode of Rut to PTP1B at an atomic level (**Fig. 7a**). The results suggest that Rut forms a hydrogen bond with the side chain of Asp48 and interacts with the side chain of Tyr46 *via* π-π stacking, and hydrophobically interacts with Arg47, Asp181, Phe182, Ser215, Ser216, Ala217, Ile219, Gly220, and Arg221 in PTP1B catalytic site (**Fig. 7a**). Moreover, the importance of Asp48 within Rut/PTP1B interaction is further validated by the reduced inhibitory potency of Rut against the D48A mutant of PTP1B (Supplementary **Fig. S4**).

On the other hand, our determined crystal structure of RXRα-LBD complexed with Rut and SRC1 indicates that Rut binds to the ligand-binding pocket (LBP) by interacting with the hydrophobic residues Val265, Leu309, Ile310, Phe313, Leu325, Leu326, Val342, Ile345, Phe346, Leu433, His435, Leu436 and Phe439 (**Fig. 7b**). Two hydrogen bonds form between the oxygen atom OAG of Rut and two nitrogen atoms of Arg316. On the other side of Rut, the oxygen atom OAH also forms one hydrogen bond with Cys432. Rut is further stabilized by the water-mediated hydrogen bonds with Leu309 and Arg316. Superposition of Rut-bound RXRα-LBD with 9-*cis*-retinoic acid-bound structure [27] shows perfect overlapping of these two agonists, thus revealing their potential similar agonistic mechanisms (**Fig. 7c**). Arg316 is found to play an essential role by forming identical hydrogen bonds with the oxygen atoms from both 9-*cis*-retinoic acid and Rut. In the current structure, Rut also activates RXRα by overturning the C-terminal helix 12, consequently recruiting the co-activator SRC1. All these observations have supported that Rut functions as an RXRα agonist, in consistent with the pertinent cell and tissue based assays.

### (+)-Rutamarin (Rut) improves glucose homeostasis and insulin sensitivity in diet-induced obese (DIO) mice

**Figure 8:**
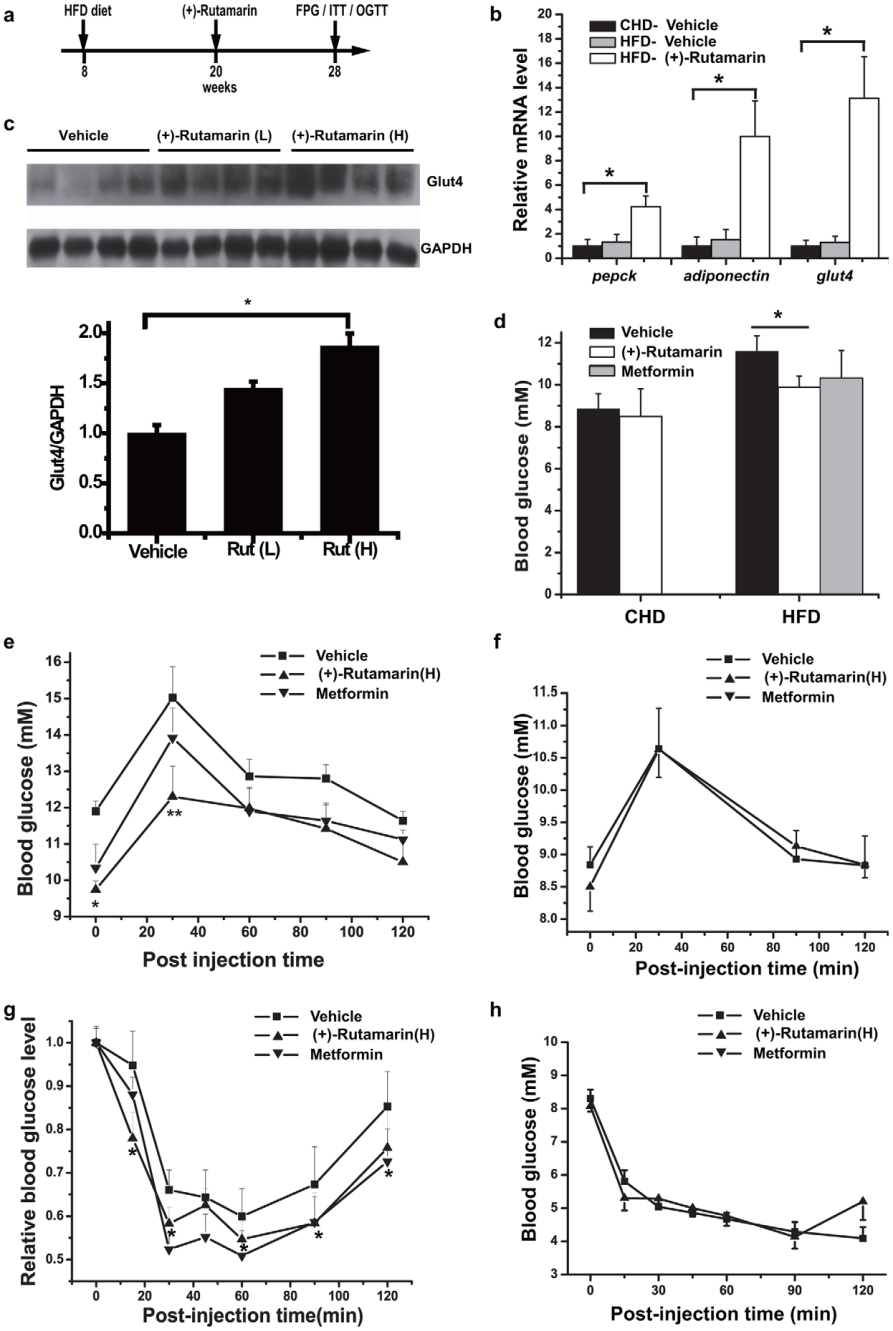
(+)-Rutamarin efficiently improves glucose homeostasis and insulin sensitivity in diet-induced obese (DIO) mice. (**a**) Scheme of C57/BL6 DIO male mice model and compound administration (HFD, high-fat diet; FPG, fasting plasma glucose; OGTT, oral glucose tolerance test; ITT, insulin tolerance test). Vehicle, Rut (10 mg/kg) or metformin (200 mg/kg) is administered daily by intraperitoneal (IP) injection for 8 weeks. (**b**) Rut (10 mg/kg) efficiently increases the mRNA levels of PPARγ:RXRα-regulated genes, including *pepck*, *GLUT4* and *adiponectin* in epididymis fat tissues (n = 7 for each group). CHD (chow diet), HFD (high-fat diet). (**c**) Rut increases GLUT4 expression in epididymis fat tissues (n = 7 for each group). L and H stand for low (2 mg/kg) and high dose (10 mg/kg) of Rut, respectively. (**d**) Rut (10 mg/kg) decreases the fasting plasma glucose (n = 7 for each group) after 8-week administration. (**e**) Rut (10 mg/kg) improves glucose disposal rate (n = 7 for each group). The oral glucose tolerance tests indicate the time course of glucose excursion following oral administration of glucose (2 g/kg) in vehicle and Rut (10 mg/kg) treated DIO mice (n = 7 for each group). (Vehicle-V, Rut-R, Metformin-M). (**f**) Rut (10 mg/kg) has no effects on glucose disposal rate in control lean mice (n = 7 for each group). Rut (10 mg/kg) increases insulin sensitivity in DIO mice (n = 7 for each group). (**g**) Insulin tolerance tests are performed in mice from each group (vehicle, 10 mg/ml Rut). Lines indicate the time courses of glucose levels after IP injection of insulin (1 U/kg). (**h**) Rut (10 mg/kg) has no effects on insulin sensitivity in control lean mice (n = 7 for each group). (▪ for vehicle-treated group, ▴ for Rut-treated group, and ▾ for metformin-treated group). Data are presented as means ± s.e.m (*P<0.05, **P<0.01).

The *in vivo* anti-hyperglycemic effects of Rut are investigated against DIO mice. In the assay, C57BL mice are fed with either regular chow diet or high-fat diet (HFD) for 3 months, and Rut (10 mg/kg), vehicle or metformin (200 mg/kg, as a positive control) are subsequently administered by intraperitoneal (IP) injection for 2 months (**Fig. 8a**). Food consumption is not significantly different between the Rut and vehicle-treated mice. The body weights of Rut and vehicle-treated mice slightly decrease in the first four weeks, and become stable in the last four weeks, which may be caused by mice in-adaption of compounds administration at the first 4 weeks. No other significant abnormal animal responses are found with Rut administration, and there is no overt toxicity in the body organs such as liver, kidney, spleen and heart, and no distinct difference in the weights of these organs between Rut-treated and control groups (Supplementary **Fig. S3c**).

As indicated in **Fig. 8b**, Rut could efficiently increase the mRNA levels of PPARγ:RXRα-regulated genes in epididymis fat tissues, including *pepck*, *GLUT4* and *adiponectin* [7], [28], [29], which suggests the potential Rut-involved activation of PPARγ:RXRα heterodimer *in vivo*. Additionally, Rut also increases GLUT4 expression in epididymis fat tissues (**Fig. 8c**). In addition, the fasting plasma glucose of Rut-treated DIO mice is significantly lower than the control obese group, while no obvious difference is observed between Rut and vehicle-treated lean mice (**Fig. 8d**). For investigation of the potential acute effect of Rut on mice, Rut (2 mg/kg) is subsequently administered by intraperitoneal (IP) injection for 2 weeks, and we find that Rut could efficiently improve fasting plasma glucose level and insulin receptor (IR) phosphorylation (Supplemental **Fig. S1f, g**).

To further explore the effects of Rut on glucose homeostasis, glucose tolerance test (GTT) is employed. As shown in **Fig. 8e**, fasting plasma glucose is much lower after glucose challenge in Rut-treated DIO mice compared with the vehicle-treated group (**Fig. 8e**), while Rut and vehicle-treated lean mice exhibit similar profiles in glucose clearance over time (**Fig. 8f**). Furthermore, the improvement of insulin sensitivity by Rut is also evaluated by insulin tolerance test (ITT). As indicated in **Fig. 8g** with the percentage of basal glucose level, Rut-treated DIO mice eliminate glucose at a faster rate compared with the vehicle-treated group, while there is no obvious difference between Rut and vehicle-treated lean mice (**Fig. 8h**).

In addition, it is tentatively suggested that the large dose of metformin (200 mg/kg) has made the similar ability in decrease of the fasting plasma glucose level to Rut (**Fig. 8g**). As published, the anti-hyperglycemic effect of metformin was due to the suppression of lipid oxidation and hepatic glucose production [30].

## Discussion

Although the detailed pathogenic mechanisms of diabetes are still elusive, impaired insulin signaling and dys-regulated gene expression have been investigated in this disease [12]. GLUT4 plays its crucial role in the rate-limiting step for cells to utilize glucose by both of its translocation and expression [1]. Inducers of GLUT4 translocation or expression were found to exert their beneficial effects on sensitizing insulin signal and recovering the dys-regulated gene expressions, eventually resulting in amelioration of metabolic syndrome [15]–[17]. Specific dual-inducers of both GLUT4 translocation and expression could be thus expected to become more potent for metabolic syndrome treatment. To date, targeting GLUT4 translocation, varied methods have been designed to screen insulin sensitizers, but most of them cannot meet the high throughput compound screening [20], [22], [31]. In the current work, we have constructed the IN Cell Analyzer 1000 based screening platform to discover the dual inducers of both GLUT4 translocation and expression. We finally determine that the natural product (+)-Rutamarin functions as this dual inducer (**Fig. 1**). It could largely enhance the insulin-stimulated glucose uptake in 3T3-L1 adipocytes as a potent insulin sensitizer (**Fig. 1h**). The *in vivo* study further indicates that Rut efficiently increases GLUT4 expression in epididymis fat tissues and improves glucose homeostasis and insulin sensitivity in DIO mice (**Fig. 8**).

Target inspection has demonstrated that targeting PTP1B is responsible for Rut stimulating GLUT4 translocation, while Rut promotes GLUT4 expression by functioning as an RXRα agonist. Enzymatic assay suggested that Rut is a competitive inhibitor of PTP1B (**Fig. 3**). Molecular modeling and site-directed mutagenesis further confirm that Rut binds to the substrate pocket of PTP1B (**Fig. 7a** **and** Supplementary **Fig. S4**). PTP1B has been proved to be a validated target against diabetes [32], and antisense oligonucleotides of PTP1B has already entered phase II clinical trials [33]. Since PTPs share highly structural conservation, selectivity and bioavailability restrictions have challenged the development of small inhibitors against PTP1B. Here Rut has been determined to exhibit good selectivity against PTP1B over other PTPs family members, highlighting its valuable potential in the discovery of drug lead compound against diabetes.

As reported, broad spectrum inhibitors of PTPs like vanadate and pervanadate, induce GLUT4 translocation by a mechanism independent of PI3-kinase, as indicated by the fact that the known PI3-kinase inhibitor wortmannin does not inhibit the pervanadate-stimulated glucose uptake [34]. Interestingly, our work demonstrates that Rut could efficiently stimulate PI3-kinase pathway, and wortmannin completely inhibits Rut or insulin-stimulated GLUT4 translocation (**Fig. 2f**), these results thus demonstrate that PI3 kinase-AKT/PKB pathway is necessary for the Rut-induced GLUT4 translocation. Compared with C2, a non-competitive inhibitor of PTP1B [18], Rut functions as a competitive PTP1B inhibitor and potently increases the insulin-stimulated phosphorylation levels of IR and AKT. In addition, the greater effects of Rut over pervandate and C2 might be tentatively ascribed to their different PTP-1B inhibition modes and the potential wortmannin-sensitive related pathways for Rut.

RXRα interacts with different nuclear receptors to form heterodimers, thus executing different functions [2]. The pleiotropic roles of RXRα have made this nuclear receptor an attractive target for drug discovery. To date, some RXRα modulators have been reported to exhibit glucose-lowering, insulin-sensitizing or anti-obesity effects [35]. Here, Rut is determined as a heterodimer-selective agonist. It activates PPARγ:RXRα but exhibits no effects on LXRα:RXRα or FXR:RXRα dimerization. Such a PPARγ:RXRα heterodimeric selectivity for Rut is expected to benefit its potential in drug development [8]. Different from rosiglitazone and pioglitazone, the two members of thiazolidinedione (TZD) PPARγ agonists with anti-hyperglycemic activity [36], Rut could increase GLUT4 expression *via* enhancing RXRα-LBD/SRC1 interaction and PPARγ:RXRα heterodimer transcriptional activity through direct binding to RXRα.

The anti-hyperglycemic effect of Rut has been investigated in DIO mice, and the greatly elevated expression of PPARγ:RXRα in Rut-treated group could be clearly determined (**Fig. 8b**). In addition, our results demonstrate that the glucose homeostasis is improved in Rut-treated DIO mice as indicated by the decreased fasting plasma glucose and enhanced glucose clearance rate in GTT assay (**Fig. 8g, e**). Moreover, Rut efficiently meliorates insulin sensitivity in 3T3-L1 adipocytes, and the insulin sensitivity of the Rut-treated animals is also statistically improved. Since GLUT4 is the principal glucose transporter responsible for glucose disposal, the Rut-induced increament of both GLUT4 translocation and expression may account for the improved glucose homeostasis in DIO mice, while the inhibition of PTP1B of Rut could benefit the improved *in vivo* insulin sensitivity. In addition, the fact that Rut functions as an RXRα agonist and selectively activates PPARγ:RXRα heterodimer may also be contributed to its anti-hyperglycemic and insulin-sensitizing effects [37], [38].

In conclusion, we have identified the natural product Rut as the first reported dual inducer of both GLUT4 translocation and expression, which can efficiently improve glucose homeostasis and insulin sensitivity in DIO mice. Further target exploration research indicates that Rut functions as both a specific PTP1B inhibitor and an RXRα agonist that selectively activates PPARγ:RXRα heterodimer. Our results have implied that Rut could serve as not only a promising lead compound for further anti-T2DM drug discovery, but also a valuable chemical probe for new GLUT4-associated pathways investigation.

## Materials and Methods

### Materials

All cell culture reagents are purchased from GIBCO and all BIAcore agents are from GE Healthcare. Insulin (cellular assay), wortmannin, penicillin-streptomycin, biotin, p-nitrophenyl-β-D-galactopyranoside (PNP-β-Gal), cytochalasin B, *p*-nitrophenyl phosphate (*p*NPP), D-glucose and Ca-pantochenate are purchased from Sigma-Aldrich. Alexa Fluor647 goat anti-mouse IgG is purchased from Molecular Probes (Invitrogen). All antibodies for western blot are from Cell Signaling Technology except anti-GAPDH antibody (KangChen, China), anti-myc antibody (Tiangen Biotech, China) and anti-GLUT4 antibody (Santa Cruz). BCA Protein Assay Kit is purchased from Beyotime (China). The Clontech MATCHMAKER Two-Hybrid System is a generous gift from Prof. Y. Gong (Shanghai Institutes for Biological Sciences, CAS, China). Dual Luciferase Assay System is purchased from Promega. 2-[^3^H]-deoxy-D-glucose is from Perkinelmer. Insulin (IP injection) is from Humulin®, Lily. High fat diet (D12492i) is purchased from Research Diets Inc. TRIzol reagent is from Generay Biotech, China. PrimeScript™ RT reagent Kit is obtained from TaKaRa, Japan. SYBR Green Real time PCR master mix is from TOYOBO, Japan. (+)-Rutamarin (Rut) is synthesized as described [39].

### Plasmids

pEGFPN1-GLUT4-myc vector is cloned from pCXN2-IRGT-myc (kindly provided by Dr. Yousuke Ebina, University of Tokushima, Japan.) with *Xho*I-*BamH*I sites, and pGL3-GLUT4-pro was described previously [40]. pGBKT7-RXRα-LBD_223–463_ and pGADT7-SRC1_613–773_ were cloned using human cDNA as templates with *EcoR*I-*Pst*I and *EcoR*I-*BamH*I sites, respectively. pSuperbasic-PPARγ and pSuperbasic-RXRα vectors were constructed by inserting the coding sequences of siRNA [41], [42] into the pSuperbasic with *Bgl*II-*Hind*III sites. The sequences were GCCCTTCACTACTGTTGAC (PPARγ) and GCACTATGGAGTGTACAGC (RXRα). pcDNA3.1-PPARγ, pcDNA3.1-RXRα, pSV-PPRE-Luc were described previously [43]. pCMX-FXR plasmid is a gift from Stefan K. Westin (X-Ceptor Therapeutics Inc., CA). pGL3-pro-RXRE-Luc vector is constructed by inserting 4 DR1 sequences with *Xho*I-*Bgl*II sites. The vector pGL3-pro-FXRE-Luc is kindly provided by Dr. Majlis Hermansson (AstraZeneca, Mölndal, Sweden), and LXRE vector is a generous gift from Prof. Charisse S. Vales (The Medicine Institute at University of California, CA). The plasmid pET15b-PPARγ-LBD_202–477_ is kindly donated by Dr. J. Uppenberg (Department of Structural Chemistry, Pharmacia and Upjohn, Sweden). The plasmid pET15b-SRC1_613–773_ is constructed with *Nde*I-*BamH*I sites, and pET22b-RXRα-LBD_223–463_ with *Nde*I-*Xho*I site, while pGEX4T-1-TC-PTP_1–288_, and pGEX4T-1-PTP1B_1–321_ with *BamH*I-*Hind*III and *BamH*I-*EcoR*I sites respectively using a PTP1B or TC45 vector as template that are kindly provided by Prof. Nicholas K. Tonks (Cold Spring Harbor Laboratory, NY). Vectors pGEX2T-LAR_1282–1888_ and pGEX2T-CD45_560–1256_ are kindly donated by Prof. Rafael Pulido (Centro de Investigación Príncipe Felipe, Spain), pGEX2T-PTPα_182–803_ is by Prof. Frank R. Jirik (University of British Columbia, Canada), and pET15b-hRXRα-LBD is by Dr. William Bourguet (Centre de Biochimie Structurale, France).

### Site-directed mutagenesis

The plasmid pGEX4T1-PTP1B (D48A) is generated by site-directed mutagenesis with pGEX4T1-PTP1B (WT) as template. The primer pairs are sense: 5′-accgaaataggtacagagccgtcagtccctttgaccatag-3′ and antisense: 5′-ctatggtcaaagggactgacggctctgtacctatttcggt-3′.

### Protein purification

The plasmids pGEX2T-PTPα, pGEX4T-TC-PTP, pGEX2T-LAR, pGEX2T-CD45, pGEX4T-PTP1B, pET15b-SRC1, pET15b-hPPARγ-LBD, and pET15b-RXRα-LBD are transformed into BL21 (DE3), respectively. The cells are grown at 37°C in LB medium. After 4 h induction with isopropyl-β-D-thiogalactopyranoside (0.5 mM, IPTG) at suitable temperature (22°C for GST-TC-PTP, GST-CD45, 37°C for GST-PTP1B, 25°C for PTPα, LAR, PPARγ, RXRα and SRC1_613–773_), the cell cultures are collected and frozen at −80°C. Purifications of GST-tagged proteins (GST-PTP1B, GST-TC-PTP, and GST-CD45) are carried out according to the manufacturer's instructions (Amersham Pharmacia), while purification of His-tagged proteins, including SRC1, PPARγ-LBD, and RXRα-LBD are processed based on the manufacturer's instructions (Novagen).

### Cell culture and differentiation

CHO-K1 and CHO-K1/GLUT4 cells are cultured in F-12 Ham medium supplemented with 10% FBS, penicillin-streptomycin (50 U/ml). HEK293 cells are cultured in Delbecco's modified Eagle's medium (DMEM) supplemented with 10% FBS, and 3T3-L1 adipocytes were cultured in DMEM supplemented with 10% FBS, penicillin-streptomycin, biotin (3.2 mM) and Ca-pantochenate (16.8 mM). All cells are cultured at 37°C in humidified atmosphere with 5% CO_2_, and the differentiation procedure of 3T3-L1 followed the classic method [44]. Briefly, two days after 100% confluence, cells are stimulated with MDI stimulus (0.5 mM Methylisobutylxanthine, 1 µM Dexamthasone and 170 nM insulin) for 3 days, and then cells are stimulated with 170 nM insulin for another 3-day.

### Construction of CHO-K1/GLUT4 stable cell lines

CHO-K1 cells are transfected with pEGFPN1-GLUT4-myc plasmid using Lipofectamine 2000, and subsequently screened with G418 (800 µg/ml). The CHO-K1/GLUT4 stable cell line is obtained by picking up clones.

### Quantification of GLUT4 translocation ratio

CHO-K1/GLUT4 cells are seeded into 96-well plate. After cells reached 100% confluence, F12 medium is changed to 3T3-L1 medium and incubated for 2 days [45]. 8-hour serum starvation later, cells are stimulated with insulin for 5 minutes, fixed with 3.7% formaldehyde for 15 minutes, and labeled with anti-myc monoclonal antibody and secondary antibody (Alexa Fluor 647 conjuncted anti-mouse antibody). Fluorescence pictures were obtained by IN Cell Analyzer 1000 Instrument (GE Healthcare) with the same exposure time. The basic unit comprises several core components including a Nikon microscope and a high-resolution CCD camera. Membrane-located GLUT4 is represented by the intensity of red fluorescence (Alex fluor 647) and normalized by the intensity of green fluorescence (GLUT4-EGFP), which is assigned to the total GLUT4 [46].

For GLUT4 translocation assay in 3T3-L1 adipocytes, the cells are fully differentiated (the 10th day after MDI stimulation), and seeded onto 24-well plates. After cultured in serum-free medium for 8 h, cells are incubated with Rut for 2 h and insulin for 10 min, and then rinsed with PBS, fixed with 4% formaldehyde for 10 min, and permeated with 0.2% Triton X-100 in PBS for 10 min. Cells are subsequently washed with PBS and 3% bovine serum albumin for 20 min, and sequentially incubated with primary antibody (Abcam GLUT4 antibody) for 1 h and secondary antibody (Alexa Fluor 488 conjuncted anti-mouse antibody) for another 1 h. Finally, the glass-slips are washed with PBS for examination using OLYMPUS FV-1000 confocal microscope. All pictures are made in the same time experiment.

### Western blot

Insulin sensitizing effects of Rut are investigated in CHO-K1 and 3T3-L1 adipocytes. After incubation with compounds in serum-free medium for indicated time, cells are stimulated with insulin (170 nM for CHO-K1 cells and 17 nM for 3T3-L1 adipocytes) for 5 min, and the stimulation is stopped by ice-cold PBS. The effect of Rut on insulin signaling is also investigated in CHO-K1 cells. In the assay, cells are stimulated with Rut (20 µM, 8 h) or insulin (170 nM, 5 min), and then lysed on ice. GLUT4 protein level is studied in the differentiated 3T3-L1 adipocytes (the 10th day after MDI stimulation). After incubation of compounds for 48 h, cells are lysed on ice. The proteins are resolved by SDS-polyacrylamide gel electrophoresis and electro-transferred to polyvinylidene difluoride membranes. The membrane is blocked by 5% skimmed milk and incubated with antibodies. Proteins are visualized by the ECL detecting system.

### 2-Deoxyglucose uptake in 3T3-L1 adipocytes

3T3-L1 adipocytes are fully differentiated (the 10th day after MDI stimulation) and seeded into 24-well plate. After incubation with compounds in serum-free medium containing 0.5% BSA for 8 hours, medium is changed to Krebs buffer containing 17 M insulin and incubated for 30 minutes. In the last 5 minutes, cells are incubated with 2-[^3^H]-deoxy-D-glucose. The glucose uptake is stopped by ice-cold PBS, and the cells are then lysed with 0.1% Triton. Finally the radioactivity is calculated by a scintillation counter. Cytochalasin B is used to measure nonspecific uptake, and the value is subtracted from all the data. Radioactivity is normalized by total protein concentration that is measured by BCA kit.

### PTP1B enzyme inhibition assay

The enzymatic activities of the recombinant PTPs are measured at 25°C using *p*NPP as substrate. The initial velocities of enzymatic reactions are determined by continuously measuring the absorbance at 405 nm every 15 s using a microplate spectrophotometer (Bio-Rad). To evaluate IC_50_ values, PTPs (0.3 µM) are incubated with compounds in reaction buffer (50 mM HEPES, pH 7.0, 1 mM EDTA, and 100 mM NaCl) for 10 min and the reactions are initiated by addition of *p*NPP (5 mM). To determine the inhibition type, each concentration of Rut (0, 50 and 75 µM) is incubated with PTP1B (0.3 µM) in reaction buffer (50 mM HEPES, pH7.0, 1 mM EDTA, and 100 mM NaCl) for 10 min and the reactions are initiated by different concentrations of *p*NPP (0.5–10 mM). The inhibition type is determined using double-reciprocal plots. In determination of the selectivity of Rut against PTPs, the result is referred as no inhibition when 100 µM Rut inhibits no enzyme activity. The same reaction systems are designed for all PTPs including PTP1B.

### Surface plasmon resonance (SPR) technology based assay

Binding affinities of Rut towards proteins are analyzed using SPR technology based Biacore 3000 instrument (GE Healthcare). In the assay, proteins are immobilized to CM5 chips using a standard amine-coupling procedure (Biacore manual). The proteins to be covalently bound to the chips are diluted in 10 mM sodium acetate buffer (pH4.5) to a final concentration of 0.10 mg/ml. Before experiments, baseline is equilibrated with a continuous flow of running buffer (10 mM HEPES, 150 mM NaCl, 3 mM EDTA, and 0.005% (v/v) surfactant P20, pH7.4) for 2 hours. Subsequently, Rut with a gradient of concentrations is injected to the channels at a flow rate of 20 µL/min for 60 seconds, followed by disassociation for 120 seconds. The 1∶1 Langmuir binding fitting model in BIAevaluation software version 3.1 (Biacore) is used to determine the equilibrium dissociation constants (*K*_D_s) of compounds. For SRC1 recruitment assay, purified SRC1_613–773_ is immobilized to a CM5 chip. Different concentrations of PPARγ-LBD or RXRα-LBD are injected to the channel at a flow rate of 20 µL/min for 60 s, followed by disassociation for 120 s. To further detect the effects of Rut on PPARγ-LBD/SRC1 or RXRα-LBD/SRC1 interactions, 1 µM PPARγ-LBD or RXRα-LBD is incubated with Rut (16.8–100 µM) for 1 hour, and then injected onto the chip.

### Transient transfection assay

HEK293 cells are transfected with luciferase reporter plus Renilla luciferase vector and other plasmids if needed for 5 hours, and cells are then incubated with indicated concentrations of compounds for another 24 h. Finally cells are lysed and luciferase activities are measured using Dual Luciferase Assay System kit. The results are presented as the fold activation relative to untreated cells after normalization with Renilla luciferase values. Each experiment is repeated at least twice, with each sample analyzed in triplicates.

### Yeast two-hybrid assay

AH109 strain is co-transfected with pGBKT7-RXRα-LBD and pGADT7-SRC1_613–773_. Cells are incubated with Rut or 9-*cis*-retinoic acid (the known RXRα agonist) for 48 hours, and α-galactosidase activity is measured as previous report [43].

### Tissue RNA extraction and real-time PCR

Four mice in each group are employed for the determination of gene expression. The total RNA is extracted from epididymis fat tissues (75 mg/mice) with TRIzol reagent, and the cDNA is synthesized using PrimeScript™ RT reagent Kit. Real-time PCR is performed using SYBR Green Real time PCR master mix on DNA Engine Opticon 2 System. The PCR cycle is 94°C for 20 seconds, 60°C for 30 seconds and 72°C for 30 seconds. The primer pairs for the related genes are described in Supplementary **Table S2**.

### Diet-induced obesity (DIO) mice model and compound administration

All animals receive humane care, and the experimental procedures are performed according to the institutional ethical guidelines on animal care. C57/BL6 male mice are obtained from Shanghai SLAC Laboratory Animal Co. Ltd, and maintained under the temperature controlled at 25°C with a 12 h light-dark cycle. Eight-week-old male mice are fed regular chow or high fat diet (HFD, D12492i, from Research Diets Inc.) for 12 weeks. The DIO mice and normal lean mice are grouped randomly, and each group contains 7 mice. Vehicle, Rut (10 mg/kg) or metformin (200 mg/kg) is administered daily by intraperitoneal (IP) injection for 8 weeks. Individual body weight and fasting plasma glucose are measured weekly. Averaged daily food intake is measured twice a week. At the termination of the study, mice are dissected, and the weight of tissues (liver, kidney, spleen, heart, perirenal fat, and epididymis fat) is determined.

The epididymis fat is lysed and the supernatant is separated by SDS-PAGE and transferred to polyvinylidene difluoride membranes (Millipore). Membranes are incubated in blocking buffer (BB) containing 5% skimmed milk with 0.5% Tween 20 in Tris-buffered saline and immunoblotted with the related antibodies overnight at 4°C in BB. After incubation, membranes are washed and incubated with horseradish peroxidase-labeled secondary antibodies (Amersham), and then the proteins are detected by SuperSignal West Pico chemiluminescent substrate (Pierce).

### Oral glucose tolerance test (OGTT) and insulin tolerance test (ITT)

Male mice treated with compounds for 8 weeks are used for oral glucose tolerance test. After 6 h fasting, plasma glucose levels are measured and glucose (2 g/kg) is loaded. Blood samples are taken from the tail vein at different time points (0, 30, 90, and 120 min). In insulin tolerance tests, after 6 h fasting, plasma glucose levels are measured and insulin (1 U/kg) is administered by IP injection. Blood samples are taken at indicated time points (0, 15, 30, 45, 60, 90, and 120 min). Blood glucose levels are measured by ACCU-CHEK Active blood sugar testmeter (Roche). Statistical evaluation is performed *via* two-way ANOVA. Significant difference at P<0.05: *, P<0.01: **.

### Molecular Modeling

Structure-based analysis against PTP1B is performed using the advanced docking program AUTODOCK 4.0 [47], [48] (PDB code: 1AAX) [49]. In the first step, a grid box is generated with 70×70×70 points altogether to encompass the whole ligand binding site. The spacing parameter is set to 0.375 Å. The affinity and electrostatic potential maps are calculated for each type of atom present in the ligand structure. Then the Lamarckian genetic algorithm (LGA) is applied to deal with the protein-ligand interactions by a newly revised scoring function that includes terms of Van der Waals, hydrogen bond, de-solvation energy, torsional free energy and unbound system's energy. The step size is set to 0.2 Å for translation and 5° for orientation and torsion. The numbers of generation, energy evaluation, and docking runs are set to 500,000, 2,500,000 and 20, respectively. Finally, the evaluation with the lowest binding energy is used to analyze ligand pose. The interaction model of ligand/PTP1B is produced using the Molecular Operating Environment (MOE) program (Chemical Computing Group, Montreal, Canada) based on the docked complex structure, and the docking approach comprises the *in silico* modeling.

### Crystallization and structure determination

The crystal of RXRα-LBD complexed with Rut and SRC1 peptide (KHKILHRLLQDSS) grows in 8% v/v Tacsimate pH8.0, 20% (w/v) PEG3350 at 4°C. Diffraction data is collected on the BL17U at Shanghai Synchrotron Radiation Facility (China) and processed with HKL2000 [50]. Phases for the structure are initially determined with Molrep and refined with Refmac5 to a resolution of 2.0 Å in the CCP4 suite [51]. Model building is performed manually with COOT [52]. The quality of the final model is checked with PROCHECK [53]. The coordinate has been deposited to the Protein Data Bank with accession numbers of 3PCU. All structural figures are prepared with PyMol [54]. The statistics of the data collection and structure refinement are summarized in Supplementary **Table S3**.

### Calculations

Data are expressed as means ± s.e.m (*P<0.05,**P<0.01). Statistical analysis is conducted using Student's t test or one-way ANOVA. OGTT and ITT data are conducted using two-way ANOVA.

## Supporting Information

Figure S1 **Dose-dependent induction of GLUT4 translocation by insulin and short-term effects of Rut.** (**a**) GLUT4 protein level is detected either in the insulin (17 nM) stimulated 3T3-L1 adipocytes for 5 minutes, or pre-treated with Rut (20 µM) for 8 hours and then stimulated with insulin (17 nM) for 5 minutes. (**b**) The serum-starved CHO-K1/GLUT4 cells are stimulated with indicated concentrations of insulin (5 min) or pervanadate (30 min). Subsequently membrane GLUT4 is determined and EC_50_ values of insulin and pervandate are fitted to 100 nM and 51 µM, respectively. (**c, d**) Effect of rut on insulin induced GLUT4 translocation and glucose uptake in time-dependent manner. (**e**) GLUT4 expression is determined by western blotting with an antibody against GLUT4 in 3T3-L1 adipocytes treated with Rut (20 µM) in time-dependent manner. (**f**) Rut (2 mg/kg) improves plasma glucose level in DIO mice (n = 7 for each group) for 2 weeks. (**g**) Insulin receptor phosphorylation level is detected in Rut-treated mice epididymis fat tissues.
(TIF)

Figure S2 **RXRα agonist 9-cis-retinoic acid (9-cis-RA) or PPARγ agonist rosglizitone (Ros) dose-dependently enhances interaction between RXRα-LBD and SRC1 or between PPARγ-LBD and SRC1.** (**a**) RXRα agonist 9-*cis*-RA or (**d**) PPARγ agonist rosglizitone (Ros) dose-dependently binds to RXRα-LBD and PPARγ-LBD, respectively. (**b**) RXRα-LBD or (**e**) PPARγ-LBD dose-dependently binds to SRC1. (**c**) 9-*cis*-RA and (**f**) rosglizitone enhances interaction between RXRα-LBD or PPARγ-LBD and SRC1.
(TIF)

Figure S3 **The effects of (+)-rutamarin administration.** (a) diet, (b) body weight and (c) main organ weights in DIO mice,average daily food intake is measured twice a week and individual body weight is measured weekly. At the termination of the study, mice are dissected, and tissue weights (Liver, kidney, spleen, heart, perirenal fat, and epididymis fat) are determined.
(TIF)

Figure S4 **Binding activity of (+)-Rutamarin with PTP1B (WT) and PTP1B (D48A).** Rut inhibits PTP1B (WT) and PTP1B (D48A) activity with IC_50_ value of 6.4 and 14.8 µM, respectively.
(TIF)

Table S1 **Association (kon) and Dissociation (koff) Rate and Equilibrium Dissociation (KD) Constants of (+)-rutamarin binding to RXRα-LBD obtained from SPR experiments.**
(TIF)

Table S2 **The Primers in Real-time PCR.**
(TIF)

Table S3 **Data collection and structure refinement statistics.**
(TIF)
