# Supplementary figures and images for: (+)-Rutamarin as a Dual Inducer of Both GLUT4 Translocation and Expression Efficiently Ameliorates Glucose Homeostasis in Insulin-Resistant Mice
Source: PLoS One. 2012 Feb 27;7(2):e31811. doi: 10.1371/journal.pone.0031811 (PMC3288053; doi:10.1371/journal.pone.0031811)

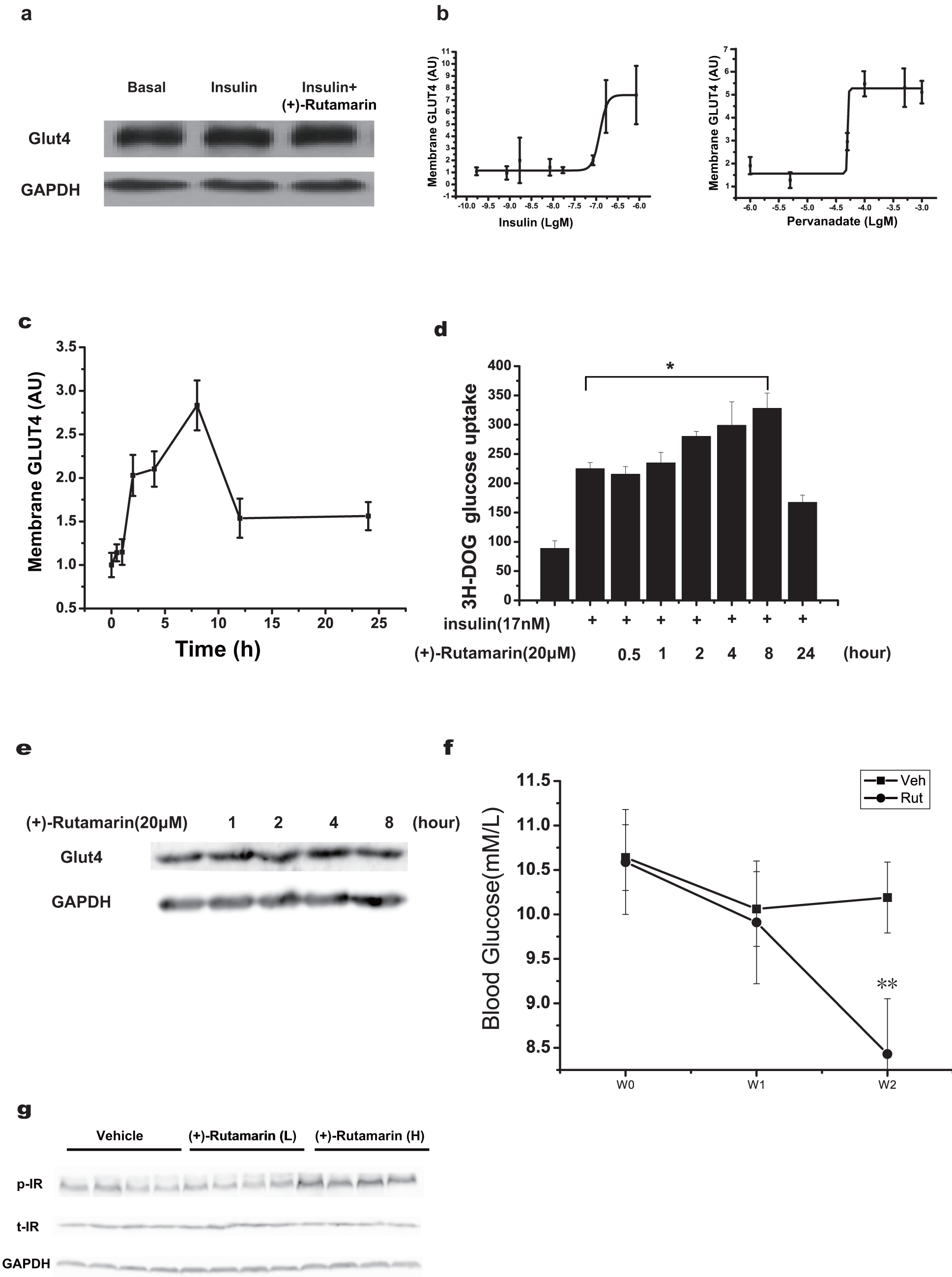

Supplement: Figure S1 — Dose-dependent induction of GLUT4 translocation by insulin and short-term effects of Rut. (a) GLUT4 protein level is detected either in the insulin (17 nM) stimulated 3T3-L1 adipocytes for 5 minutes, or pre-treated with Rut (20 µM) for 8 hours and then stimulated with insulin (17 nM) for 5 minutes. (b) The serum-starved CHO-K1/GLUT4 cells are stimulated with indicated concentrations of insulin (5 min) or pervanadate (30 min). Subsequently membrane GLUT4 is determined and EC50 values of insulin and pervandate are fitted to 100 nM and 51 µM, respectively. (c, d) Effect of rut on insulin induced GLUT4 translocation and glucose uptake in time-dependent manner. (e) GLUT4 expression is determined by western blotting with an antibody against GLUT4 in 3T3-L1 adipocytes treated with Rut (20 µM) in time-dependent manner. (f) Rut (2 mg/kg) improves plasma glucose level in DIO mice (n = 7 for each group) for 2 weeks. (g) Insulin receptor phosphorylation level is detected in Rut-treated mice epididymis fat tissues. (TIF) [file pone.0031811.s001.tif]

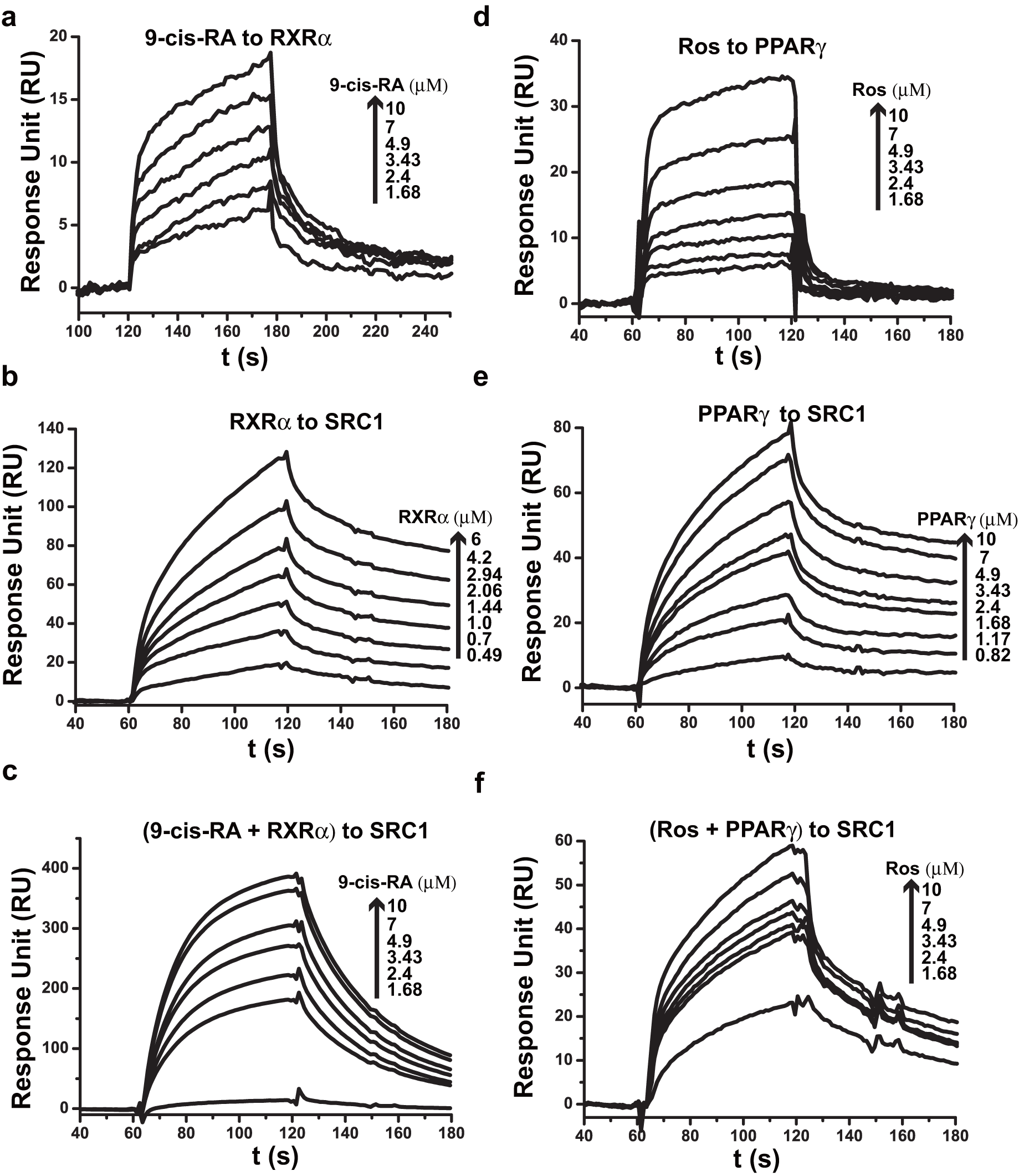

Supplement: Figure S2 — RXRα agonist 9-cis-retinoic acid (9-cis-RA) or PPARγ agonist rosglizitone (Ros) dose-dependently enhances interaction between RXRα-LBD and SRC1 or between PPARγ-LBD and SRC1. (a) RXRα agonist 9-cis-RA or (d) PPARγ agonist rosglizitone (Ros) dose-dependently binds to RXRα-LBD and PPARγ-LBD, respectively. (b) RXRα-LBD or (e) PPARγ-LBD dose-dependently binds to SRC1. (c) 9-cis-RA and (f) rosglizitone enhances interaction between RXRα-LBD or PPARγ-LBD and SRC1. (TIF) [file pone.0031811.s002.tif]

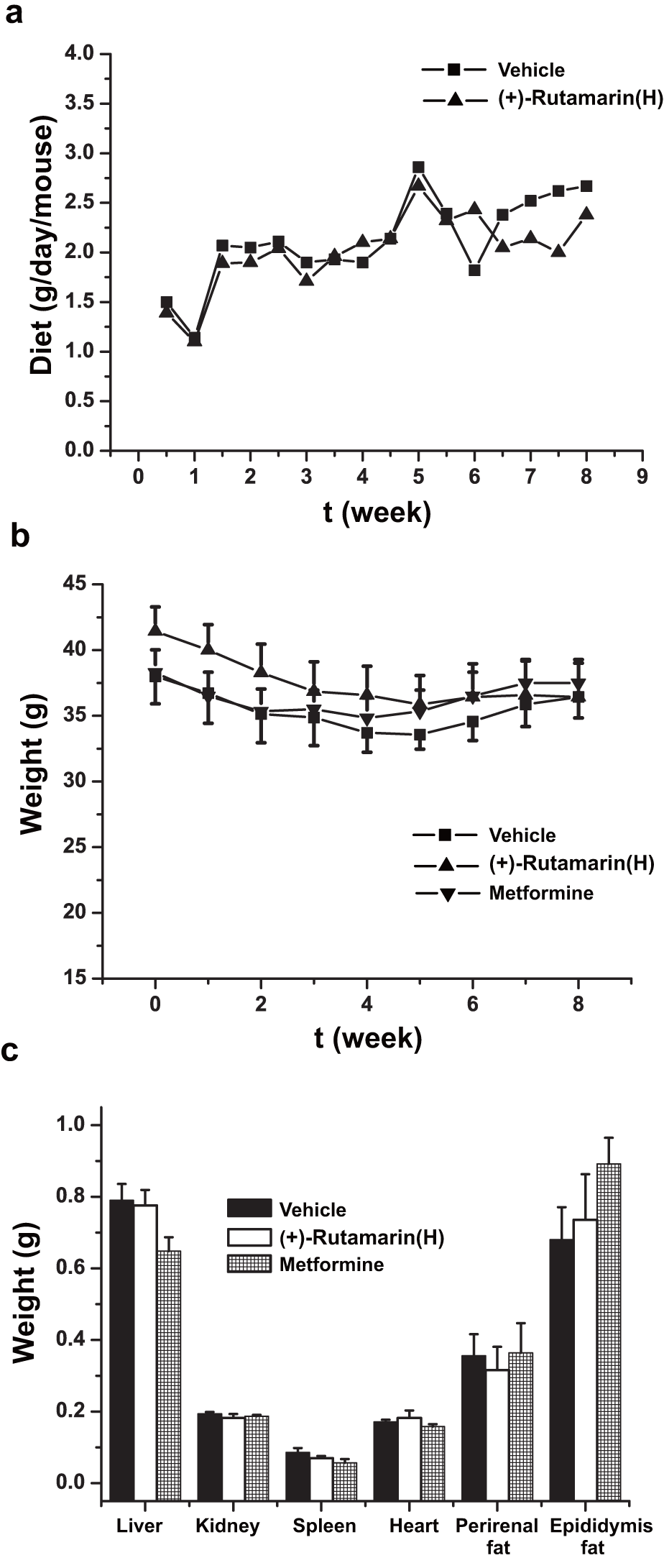

Supplement: Figure S3 — The effects of (+)-rutamarin administration. (a) diet, (b) body weight and (c) main organ weights in DIO mice,average daily food intake is measured twice a week and individual body weight is measured weekly. At the termination of the study, mice are dissected, and tissue weights (Liver, kidney, spleen, heart, perirenal fat, and epididymis fat) are determined. (TIF) [file pone.0031811.s003.tif]

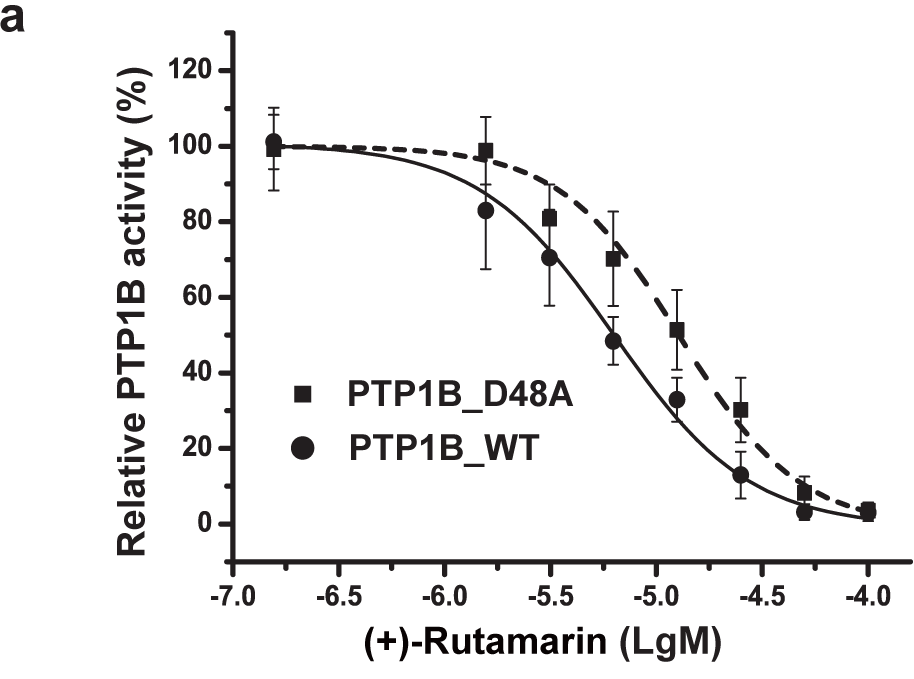

Supplement: Figure S4 — Binding activity of (+)-Rutamarin with PTP1B (WT) and PTP1B (D48A). Rut inhibits PTP1B (WT) and PTP1B (D48A) activity with IC50 value of 6.4 and 14.8 µM, respectively. (TIF) [file pone.0031811.s004.tif]

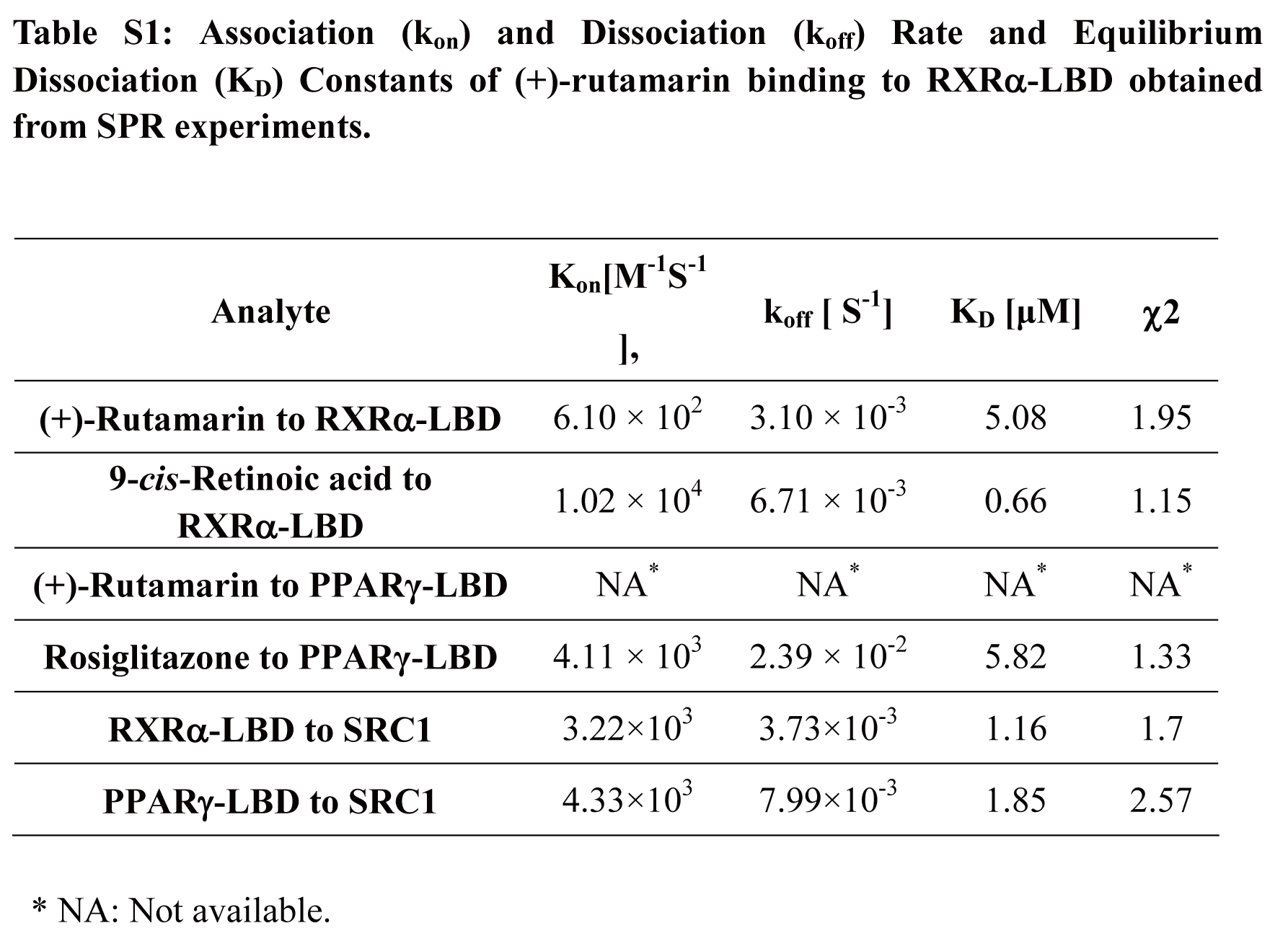

Supplement: Table S1 — Association (kon) and Dissociation (koff) Rate and Equilibrium Dissociation (KD) Constants of (+)-rutamarin binding to RXRα-LBD obtained from SPR experiments. (TIF) [file pone.0031811.s005.tif]

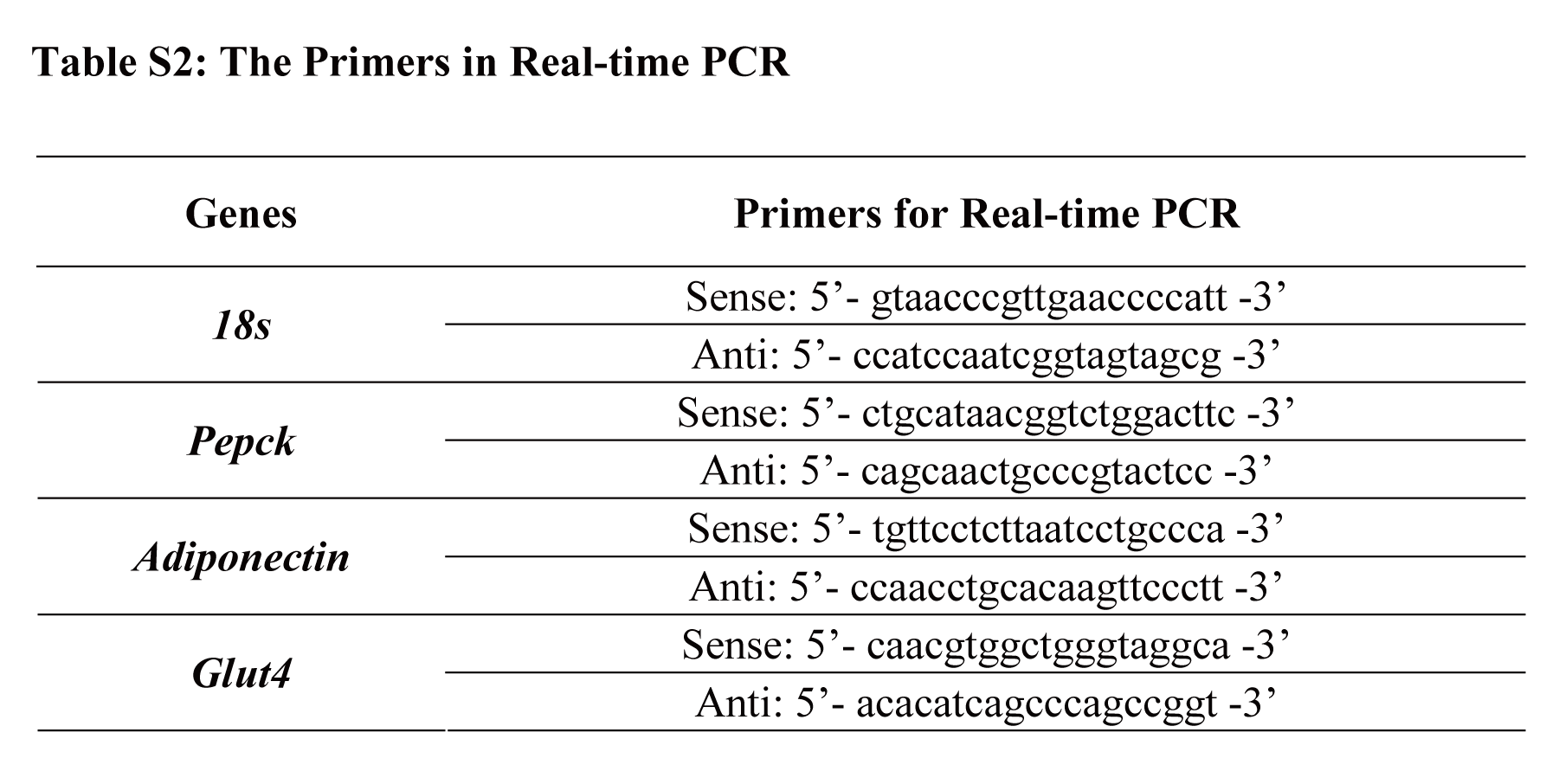

Supplement: Table S2 — The Primers in Real-time PCR. (TIF) [file pone.0031811.s006.tif]

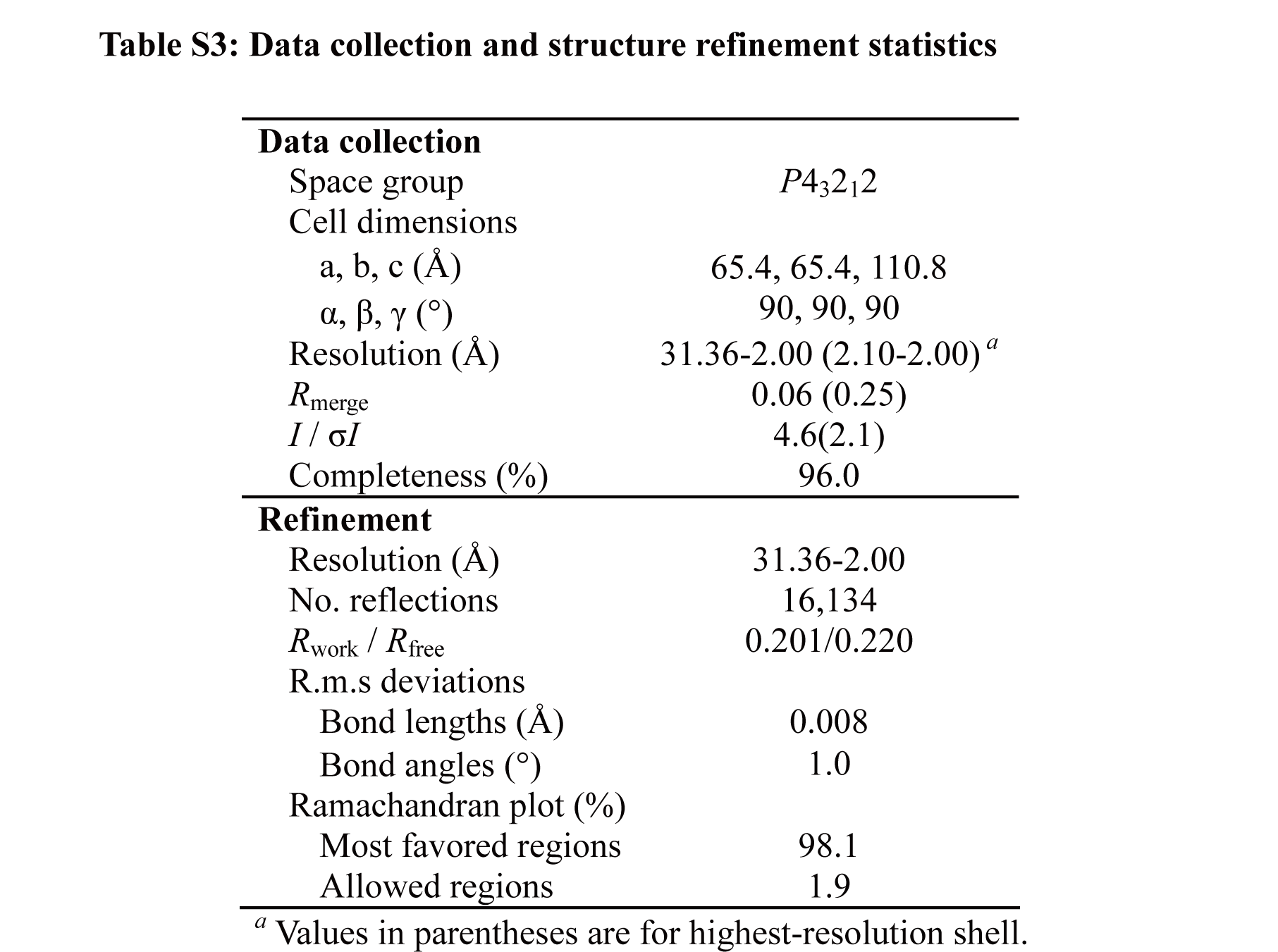

Supplement: Table S3 — Data collection and structure refinement statistics. (TIF) [file pone.0031811.s007.tif]
